# Complex Self-Organization in *n*-Alkylammonium Sulfobetaine Zwitterions with High Thermal Stabilities and High Expansion Coefficients

**DOI:** 10.1021/acs.langmuir.4c02892

**Published:** 2025-02-11

**Authors:** Alyna Lange, Lea Holtzheimer, Coby Clarke, Andreas F. Thünemann, Andreas Taubert

**Affiliations:** † Institute of Chemistry, University of Potsdam, Karl-Liebknecht-Straße 24-25, D-14476 Potsdam-Golm, Germany; ‡ GSK Carbon Neutral Laboratory, Jubilee Campus, The University of Nottingham, Nottingham NG7 2GA, U.K.; § Bundesanstalt für Materialforschung und -prüfung, Unter den Eichen 87, D-12205 Berlin, Germany

## Abstract

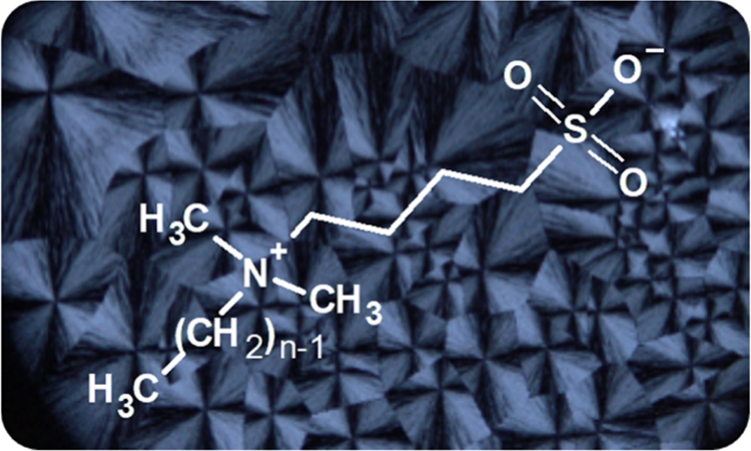

Sulfobetaine zwitterions made from *n*-alkyl dimethylamines and butanesultone yield a series of *n*-alkylammonium sulfobetaine zwitterions with complex self-organization behavior. The compounds are thermally quite stable and the length of the alkyl chain directly affects all phase transition temperatures of the compounds: the longer the alkyl chain, the higher the transition temperature. All compounds exhibit lamellar order and the different phases are characterized by a lower temperature orthorhombic and a higher temperature hexagonal in-plane order. The phase transition from the orthorhombic to the hexagonal phase is always associated with an increase of the long period. The phase transition is also associated with a rather high thermal expansion coefficient.

## Introduction

Organic salts in which anion and cation are covalently tethered are called zwitterions (ZIs). These compounds usually show physicochemical properties like higher melting temperatures that are quite different when compared to conventional organic salts (where the anion and the cation exist as two independent units).^1−4^ Some ZIs even show melting points very close to their decomposition temperatures.^2^ Many zwitterions consist of an imidazolium-, ammonium- or phosphonium unit as the positively charged moiety and a negatively charged sulfonate group that is separated by a spacer from the cationic part. The result of this covalent connection between anion and cation generates molecules with rather large dipole moments.^1,5^

One important aspect that has been studied for zwitterions is their use as surfactants and their role in the stabilization of micellar aggregates.^6−8^ Zwitterionic moieties or zwitterionic polymers can stabilize micelles at quite low critical micelle concentrations (CMC). Different drugs can then be encapsulated into the resulting micelles, opening up interesting pathways for e.g., drug delivery.^9,10^

Zwitterions are therefore also interesting compounds from a biological research standpoint. Due to their overall antifouling nature and their excellent inhibition of plasma protein adsorption, ZIs have attracted growing interest for application in blood-inert surfaces, as they can improve hemocompatibility of the surfaces of polymeric substrates.^11,12^ The antifouling nature of ZIs is therefore beneficial for implants as ZIs show high resistance to the attachment of proteins, cells and bacteria. Therefore, harmful anaphylactic responses, clotting around implants or bacterial infections associated with the use of biomaterial implants are reduced.^10,13−16^ For this last application the ZIs are usually combined with or coated on polymers such as methacrylates, resulting in high hydration capacity and neutrally charged surfaces.^12−14,17−19^ Another application that benefits from the antifouling aspect of the ZIs can be found in the treatment and reuse of wastewater, for which the ZIs are again integrated onto membrane surfaces to prevent biofilm formation.^20^

Besides biological and medical uses, zwitterions have also been investigated for various applications such as electrolyte materials in batteries.^4,5^ Here the advantage of ZIs vs other electrolytes lies in the neutral net charge of the ZI, resulting in no ion migration of the zwitterion itself. Combined with e.g., lithium salts ZIs can improve efficient transport of mobile ions while the ZIs themselves do not migrate under a potential gradient.^3−5^ Moreover, combined with other salts or acids, ZIs can form ionic liquids (ILs), which have a considerably lower melting point than their respective ZIs, or even ionic liquid crystals (ILCs), which show anisotropic ionic conductivity due to the formation of highly organized mesophases.^1,21−23^

**Figure 1:**
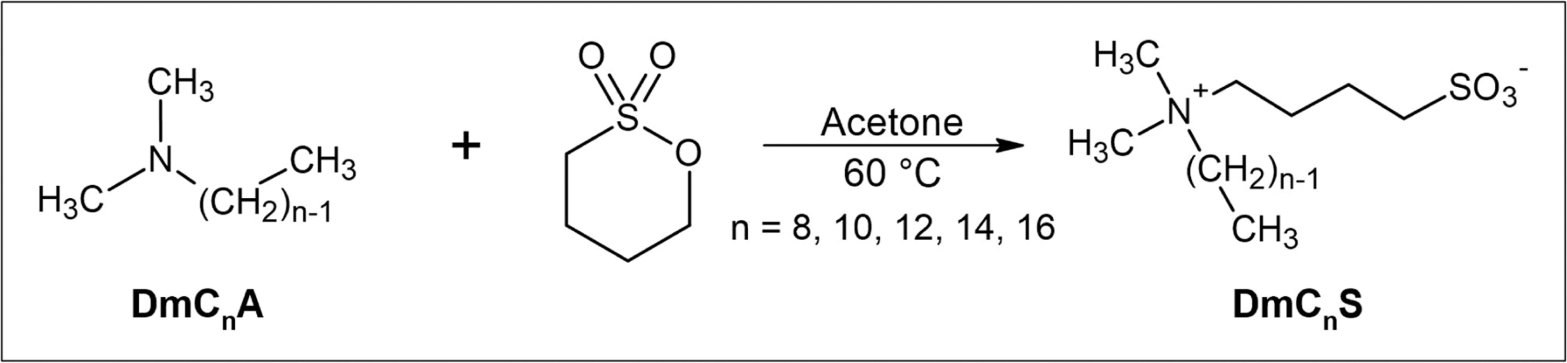
Synthesis of the zwitterions.

The current study expands the previous work by using dimethylalkylamines as the starting amines for ZI synthesis. Synthesis follows a rather straightforward process outlined in Figure 1.^22−25^ Such compounds have already been synthesized by other groups, however, they were mostly investigated under biological aspects.^3,26−29^ In addition, Mathis et al. demonstrated that amphiphilic zwitterions based on dimethylammonioalkoxydicyanoethenolates with varying chain lengths exhibit liquid crystal (LC) behavior.^30^ In this work, we expand the pool of zwitterionic liquid crystals (ZILCs) and demonstrate that dimethylalkylamines are readily available starting materials for the synthesis of sulfobetaine ZIs with interesting thermal stability and phase behavior.

## Experimental Section

### Materials

N,N-dimethyloctylamine (purity not specified by supplier, Sigma-Aldrich), N,N-dimethyldecylamine (98%, Sigma-Aldrich), N,N-dimethyldodecylamine (≥90%, TCI), N,N-dimethyltetradecylamine (≥95%, Sigma-Aldrich), N,N-dimethylhexadecylamine (≥95%, Sigma-Aldrich), 1,4-butanesultone (99%, abcr), acetone (for synthesis, Roth) were used as received.

### Preparation of Zwitterions

All ZIs were synthesized following a previously published protocol.^22,24^ The amine was filled into a two-neck round-bottom flask and stirred with a magnetic stir bar at room temperature. An equimolar amount of 1,4-butanesultone was slowly added via a syringe. After complete addition, the mixture was heated to 60 °C. When the formation of a white solid was observed, acetone was added and the reaction mixture was left to reflux for several days until sufficient amounts of precipitate were obtained. Upon cooling to room temperature the solid was filtered, washed with cold acetone (3x) and dried via rotary evaporator.
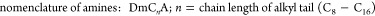

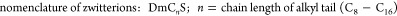


The water content of the ZIs calculated from the elemental analysis can be found in Table S1.

DmC_8_S: yield: 79.4%; ESI-MS: 294.210 g/mol (molecular mass + H^+^); elemental analysis: exp. (calc) [%]: C: 56.68 (57.30), H: 11.56 (10.65), N: 4.71 (4.77), S: 11.29 (10.93); ^1^H NMR (400 MHz, D_2_O): δ [ppm]: 0.72–0.89 (m, 3 H) 1.10–1.38 (m, 10 H) 1.61–1.99 (m, 6 H) 2.87 (t, 2 H) 2.99 (s, 6 H) 3.15–3.33 (m, 4 H). ^13^C NMR (101 MHz, D_2_O): δ [ppm]: 13.37 (s) 20.85 (s) 21.11 (s) 21.76 (s) 21.96 (s) 25.41 (s) 28.10 (s) 30.94 (s) 49.98 (s) 50.50 (s) 63.16 (s) 64.24 (s).

DmC_10_S: yield: 89.5%; ESI-MS: 322.240 g/mol (molecular mass + H^+^); elemental analysis: exp. (calc) [%]: C: 59.24 (59.77), H: 11.46 (10.97), N: 4.39 (4.36), S: 10.29 (9.97); ^1^H NMR (400 MHz, D_2_O): δ [ppm]: 0.87 (m, 3 H) 1.21–1.43 (m, 14 H) 1.66–1.97 (m, 6 H) 2.92 (t, 2 H) 3.06 (s, 6 H) 3.21–3.38 (m, 4 H). ^13^C NMR (101 MHz, D_2_O): δ [ppm]: 13.73 (s) 20.95 (s) 21.26 (s) 22.02 (s) 22.38 (s) 25.78 (s) 28.61 (s) 28.97 (s) 29.03 (s) 29.12 (s) 31.60 (s) 50.06 (s) 50.77 (s) 63.04 (s) 63.88 (s).

DmC_12_S: yield: 91.5%; ESI-MS: 350.273 g/mol (molecular mass + H^+^); C: 60.76 (61.84), H: 10.76 (11.25), N: 4.18 (4.01), S: 9.74 (9.17); ^1^H NMR (400 MHz, D_2_O): δ [ppm]: 0.91 (t, 3 H) 1.23–1.56 (m, 18 H) 1.69–2.03 (m, 6 H) 2.95 (t, 2 H) 3.13 (s, 6 H) 3.29–3.46 (m, 4 H). ^13^C NMR (101 MHz, D_2_O): δ [ppm]: 13.95 (s) 21.12 (s) 21.45 (s) 22.36 (s) 22.71 (s) 26.22 (s) 29.18 (s) 29.55 (s) 29.68 (s) 29.78 (s) 29.86 (s) 29.91 (s) 32.04 (s) 50.20 (s) 50.87 (s) 63.23 (s) 63.88 (s).

DmC_14_S: yield: 86.4%; ESI-MS: 378.306 g/mol (molecular mass + H^+^); C: 62.25 (63.61), H: 11.10 (11.48), N: 3.95 (3.71), S: 9.79 (8.49); ^1^H NMR (400 MHz, D_2_O): δ [ppm]: 1.03–1.17 (m, 3 H) 1.31–1.77 (m, 22 H) 1.87–2.28 (m, 6 H) 3.18 (t, 2 H) 3.37 (s, 6 H) 3.49–3.74 (m, 4 H). ^13^C NMR (101 MHz, D_2_O): δ [ppm]: 13.92 (s) 21.08 (s) 21.46 (s) 22.37 (s) 22.71 (s) 26.25 (s) 29.24 (s) 29.59 (s) 29.76 (s) 29.89 (s) 29.94 (s) 30.04 (s) 32.05 (s) 50.17 (s) 50.88 (s) 63.16 (s) 63.76 (s).

DmC_16_S: yield: 74.7%; ESI-MS: 406.336 g/mol (molecular mass + H^+^); C: 64.62 (65.13), H: 10.84 (11.68), N: 3.68 (3.45), S: 9.38 (7.9); ^1^H NMR (400 MHz, D_2_O): δ [ppm]: 0.95–1.09 (m, 3 H) 1.23–1.77 (m, 25 H) 1.78–2.18 (m, 6 H) 3.06–3.12 (m, 2 H) 3.27 (s, 6 H) 3.40–3.64 (m, 4 H). ^13^C NMR (101 MHz, D_2_O): δ [ppm]: 14.07 (s) 21.33 (s) 21.65 (s) 22.61 (s) 22.87 (s) 26.48 (s) 29.48 (s) 29.79 (s) 30.00 (s) 30.15 (s) 30.32 (s) 30.35 (s) 32.24 (s) 50.41 (s) 50.87 (s) 63.68 (s) 64.22 (s).

### Characterization and Instrumentation

#### Nuclear Magnetic Resonance Spectroscopy

^1^H NMR spectra were recorded on a Bruker Advance 400 MHz Spectrometer in D_2_O at room temperature. For the longer chained compounds (C_12_–C_16_ chains) small amounts of NaCl were added to the solution to improve solubility.

#### Spectroscopy

Infrared (IR) spectra were recorded using the attenuated total reflection (ATR) mode on a Thermo Scientific NICOLET iS5 with ID7 ATR probe head. Spectra were taken from 500 to 4000 cm^–1^ with a resolution of 2 cm^–1^ and 64 scans per measurement.

#### Mass Spectrometry (MS)

Electro spray ionization (ESI) MS measurements were done on a ESI-Q-TOF_micro_ with methanol used as solvent for all samples.

#### Thermal Analysis

Simultaneous thermogravimetric analysis-differential thermal analysis (TGA-DTA) experiments were done on a PerkinElmer TGA 4000 thermal balance from 30 to 600 °C with a heating rate of 10 K/min in air in aluminum oxide crucibles with lids. The lids were used to avoid moisture uptake before the measurements, but they also limit the measurement temperature to 600 °C. Differential scanning calorimetry (DSC) measurements were done on a Netzsch Polyma DSC 214. DSC traces were recorded from −100 to 200 °C using liquid nitrogen cooling and a heating rate of 10 °C/min. Heating and cooling cycles were repeated three times for reproducibility. The second heating curve was used to determine the glass transition temperature *T*_g_ and the melting temperature *T*_m_ (peak onset) and. In some cases *T*_m_ and the onset of the decomposition temperature *T*_on_ (5% mass loss) were very close to one another. In these cases *T*_m_ was approximated from heating runs during polarized optical microscopy (POM) observations and the DTA curve obtained during the TGA/DTA measurements. In these cases, data from the first heating runs were used.

#### Polarized Optical Microscopy

Optical microscopy was done on an Olympus BX53 M microscope equipped with an Olympus SC50 digital camera and a magnification of 4×. The temperature was controlled via a HS1 Hot Stage Controller and a HS82 Hot Stage by Mettler Toledo. The ZIs were dissolved in methanol and dried on a microscopy slide. The dry deposits were then heated to the respective transition temperatures identified in the DSC experiments.

#### Small-Angle X-ray Scattering

SAXS measurements were performed in a solid sample holder with a Kratky-type instrument (SAXSess, Anton Paar, Austria) in a temperature range of 20 ± 1 to 280 ± 1 °C. The SAXSess has a low sample-to-detector distance of 0.309 m, which is appropriate for investigation of samples with low scattering intensity. Each sample was measured as produced for 300 s (30 measurements of 10 s each) at every temperature. The scattering vector *q* is defined in terms of the scattering angle 2θ and the wavelength λ of the radiation (λ = 0.154 nm): thus *q* = 4π/λ sin(θ). Deconvolution (slit length desmearing) of the SAXS curves was performed with the SAXS-Quant software (Anton Paar, Austria).

## Results and Discussion

Consistent with previous studies the ZIs shown in Figure 1 have been obtained in good yields on a multigram scale. The interesting and somewhat surprising aspect, however, is that these zwitterions have never been studied for their physical and structural properties other than a very basic characterization. The current study therefore adds quite some new information on the thermal and phase behavior of these interesting substances. A special focus of this study lies on the TGA/DTA and DSC investigation along with an extensive X-ray scattering study of the phase transitions in these ZIs along with a correlation between phase behavior and molecular composition, i.e., alkyl chain length.

**Figure 2:**
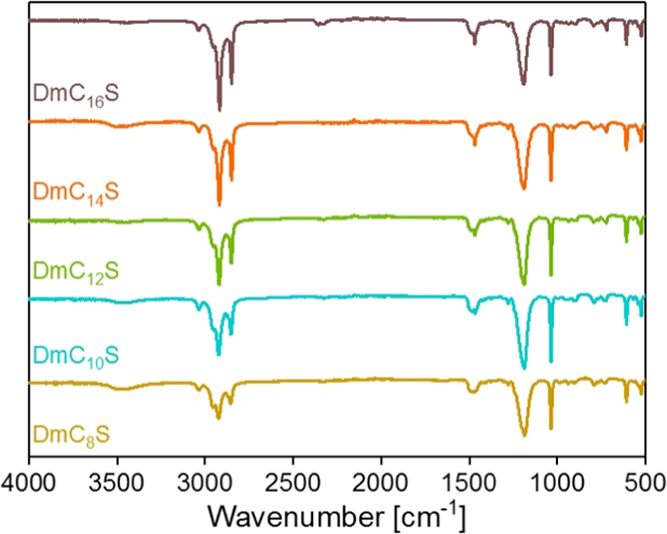
IR spectra of ZIs.

Attenuated total reflection infrared (ATR-IR) spectra of the zwitterions show typical bands associated with compounds based on alkylammonium sulfobetaines (Figure 2). A small band around 3030 cm^–1^ is associated with out-of-phase stretching vibrations of the methyl groups attached to the nitrogen. Strong bands between 3035 and 2850 cm^–1^ originate from aliphatic C–H-stretching vibrations of the alkyl chains. CH_3_ out-of-phase and CH_2_ bending vibrations can be seen around 1470 cm^–1^. The pronounced bands from 1280 to 1180 cm^–1^ arise from SO_3_-asymmetric stretching vibrations and therefore show the presence of sulfonate groups. These can also be identified via the SO_3_-symmetric stretching bands at 1040–1030 cm^–1^ and the SO_3_-bending vibrations observed from 570 to 520 cm^–1^. The band at 1179 cm^–1^ can also be associated with C–N stretching vibrations. The presence of both sulfonate and alkylammonium in the zwitterionic compound is also confirmed by the presence of bands between 760 and 720 cm^–1^. These can be assigned to C–S-stretching of the ionic headgroup and in-phase rocking of the CH_2_ groups in the alkyl chains.^31^

**Figure 3:**
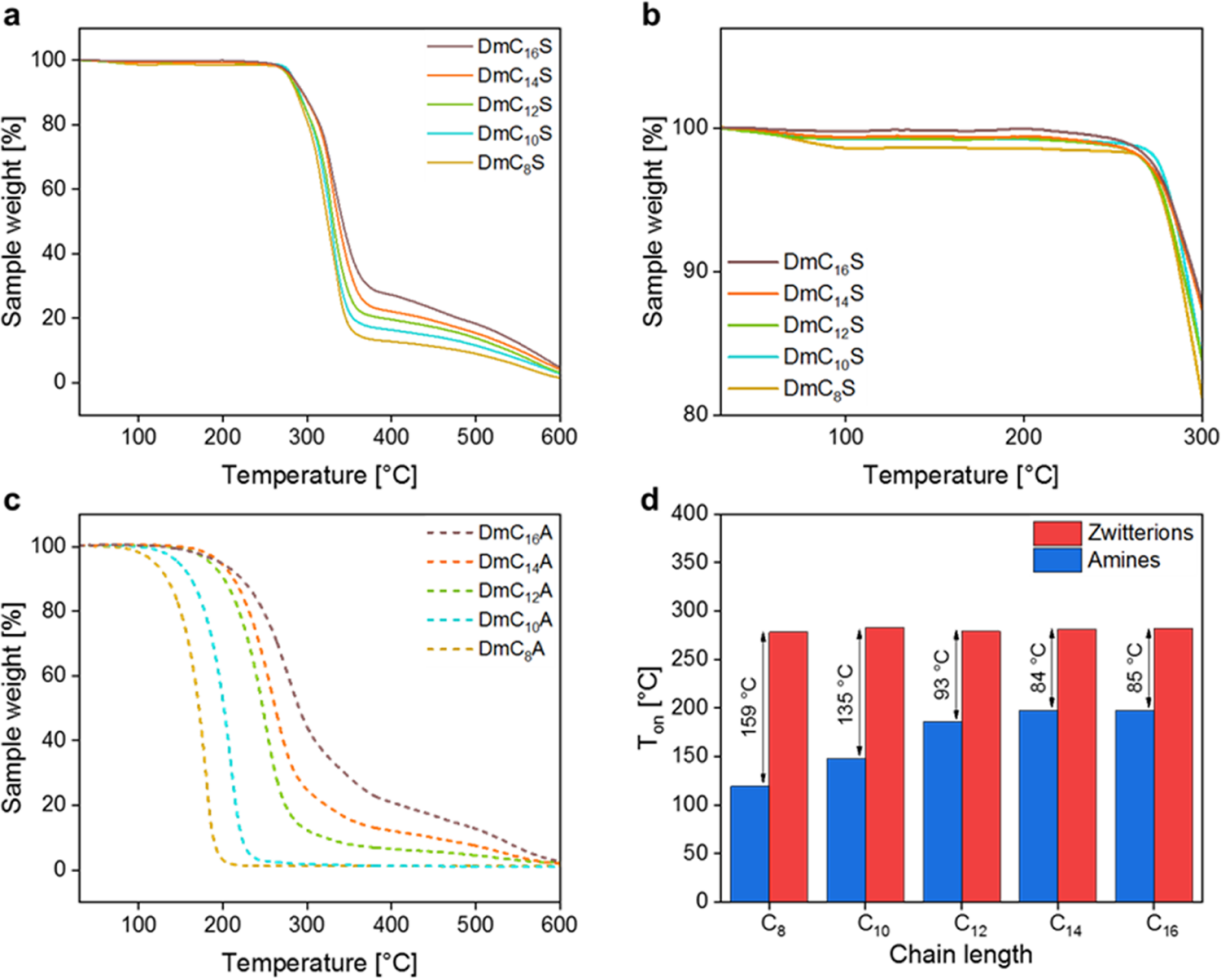
TGA data of ZIs and precursor amines**:** (a) TGA curves of ZIs up to 600 °C, (b) TGA curves of ZIs up to 300 °C, (c) TGA curves of precursor amines up to 600 °C, and (d) comparison of *T*_on_.

Phase transitions and thermal stabilities of the zwitterions were investigated with differential scanning calorimetry (DSC) and thermogravimetric analysis (TGA), respectively. Figure 3 and Table 1 show the TGA curves obtained for the ZIs and starting amines and some selected thermal data extracted from the TGA experiments.

**Table 1:** Thermal Behavior of ZIs and Precursor Amines^a^

thermal properties: amines			thermal properties: ZIs				
	*T*_600_ [%]	*T*_on_ [°C]		*T*_150_ [%]	*T*_600_ [%]	*T*_on_ [°C]	Δ*T*_on_ [°C]
DmC_16_A	97.3	197.3	DmC_16_S	0.1	95.1	282.3	85.0
DmC_14_A	97.7	197.3	DmC_14_S	0.6	95.9	281.2	83.9
DmC_12_A	96.9	185.6	DmC_12_S	0.8	96.9	279.0	93.4
DmC_10_A	99.0	147.6	DmC_10_S	0.7	97.2	282.6	135.0
DmC_8_A	98.6	119.4	DmC_8_S	1.3	98.5	278.0	158.6

a *T*_on_: onset decomposition temperature at 5% weight loss; Δ*T*_on_: difference between *T*_on_ of the ZI and the respective amine, Δ*T*_on_ = *T*_on_ (ZI) – *T*_on_ (amine); *T*_150_: weight loss in % at 150 °C; *T*_600_: weight loss in % at 600 °C.

All ZIs show a decomposition temperature *T*_on_ around 280 °C, which is in the range of *T*_on_ for other ZIs. Ohno and co-workers reported that the decomposition temperatures of alkylimidazolium or ammonium-based ZIs range between 280 and 320 °C.^3^ Narita et al. reported another imidazolium-based sulfobetaine ZI with a decomposition temperature of 321 °C and Yoshizawa-Fujita described about the same *T*_on_s for similar imidazolium-based ZIs.^2,32^ ZIs based on dimethylammonioalkoxyldicyanoethenolates as published by Mathis et al. showed *T*_on_ between 200 and 230 °C depending on the length of the alkyl chain.^30^ Wedge-shaped ZIs as described by Soberats et al. exhibited decomposition temperatures from 190 to 220 °C after forming LC phases at lower temperatures.^33^

For the ZIs investigated in this study, there does not seem to be a general trend with respect to chain length and *T*_on_ when comparing the TGA data. An interesting observation can, however, be made in connection with the decomposition steps. As the compounds from DmC_8_S to DmC_14_S show a two-step process first step with weight loss up to 85% and second step with constant weight loss up to 600 °C. In contrast, DmC_16_S shows a three-step weight loss, starting with a first step with a weight loss of around 70% and then two smaller, less pronounced steps (10% and 16%). All ZIs show almost complete decomposition at 600 °C. As *T*_on_ does not show significant differences, we must assume that the chain length of the ZIs does *not* have an influence on *T*_on_.

In contrast to *T*_on_ there *is* a general trend regarding chain length and weight loss. The first weight loss between 280–380 °C and the overall weight loss increases with decreasing chain length. This might be due to the decomposition process of the compounds. Quaternary ammonium compounds can undergo Hoffmann elimination, which leads to an amine and alkene.^34,35^ If the decomposition is a Hoffmann elimination TGA data show that there is a bigger mass loss for the shorter chained ZIs. This is consistent with the molar masses and subsequent volatility of the respective alkenes: shorter chains will lead to smaller (and hence more volatile) fragments.

This interpretation is supported by the TGA data of the precursor amines where the stability toward degradation or vaporization increases with chain length (that is, the smaller the molecular weight of the amine, the lower the *T*_on_). As an example, DmC_8_A is thermally significantly less stable and degradation is already complete at around 200 °C, vs the 600 °C of DmC_16_A (Figure 3c).

In comparison, all ZIs are thermally more stable than their respective amines. Moreover, the difference between *T*_on_ of the ZIs and the starting amines is largest for the shortest chain length C_8_. That is, the longer the chain length the closer *T*_on_ of the amine and *T*_on_ of the ZI are (Figure 3d and Table 1).

The thermal behavior of the five zwitterions was further investigated by differential scanning calorimetry (DSC) and polarized optical microscopy (POM).

**Figure 4:**
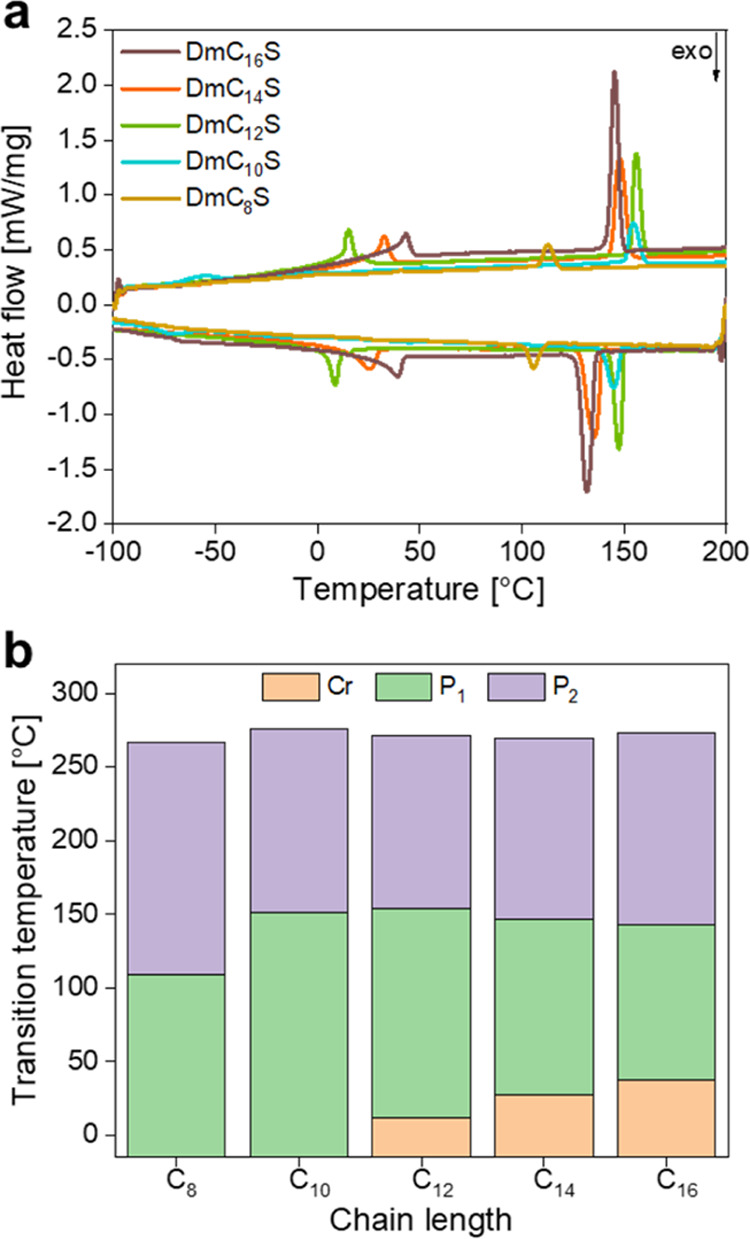
(a) 2nd heating and cooling DSC traces, and (b) graphic representation of ZI transition temperatures. P_1_ and P_2_ indicate the two lower and higher temperatures phases, respectively. As shown in the remainder of the text an unambiguous assignment of the phases is very difficult and we do hence not call them crystalline or liquid crystalline phase.

In the DSC thermograms (Figure 4a) we can identify two groups of ZIs. The longer chained compounds (DmC_12_S - DmC_16_S) show two distinct endothermic peaks on heating and cooling–the first one (*T*_1_) between 11–40 °C and the second one (*T*_2_) around 150 °C, while the shorter DmC_10_S and DmC_8_S only show *T*_2_ (see Figure S1). On heating, *T*_1_ is lower for DmC_12_S and DmC_14_S (compounds with shorter alkyl chains) than it is for DmC_16_S. This transition can be attributed to the melting of the terminal methylene groups of the long alkyl chain and the corresponding transition into the LC_1_ phase. This is qualitatively similar to other ionic compounds where the chain length directly affected the crystallinity of the respective compounds: short chains (C_8_, C_10_) showed no to very low crystallinity while longer chains led to more crystalline solids.^36−38^ The trend toward lower transition temperatures for shorter alkyl chains (C_12_ < C_14_ < C_16_) is hereby also consistent with the aforementioned literature data. The temperature windows for the mesophases can again be seen in Figure 4b. Overall, the shorter chained ZIs only show *T*_2_ as a clearly visible process. However, DmC_8_S and DmC_10_S ZIs show a range of other, broad transitions at lower temperatures that can possibly be described as glass transitions but could possibly also be assigned to other processes.

Additional DSC experiments at different heating rates show that the signal for *T*_2_ (for DmC_10_S – DmC_16_S) consists in fact of two very closely overlapping signals (Supporting Information, Figure S2). At lower heating rates (2 K/min) only one relatively sharp signal is observed, while at higher heating rates (20 K/min), a broadening of this signal and an additional shoulder toward higher temperatures are visible. These data indicate that possibly there are two phase transitions very close together. This could be due to two different polymorphs with similar stabilities, similar to Gustavsson et al.^39^ With our existing methods these two phase transitions could however not be resolved.

**Figure 5:**
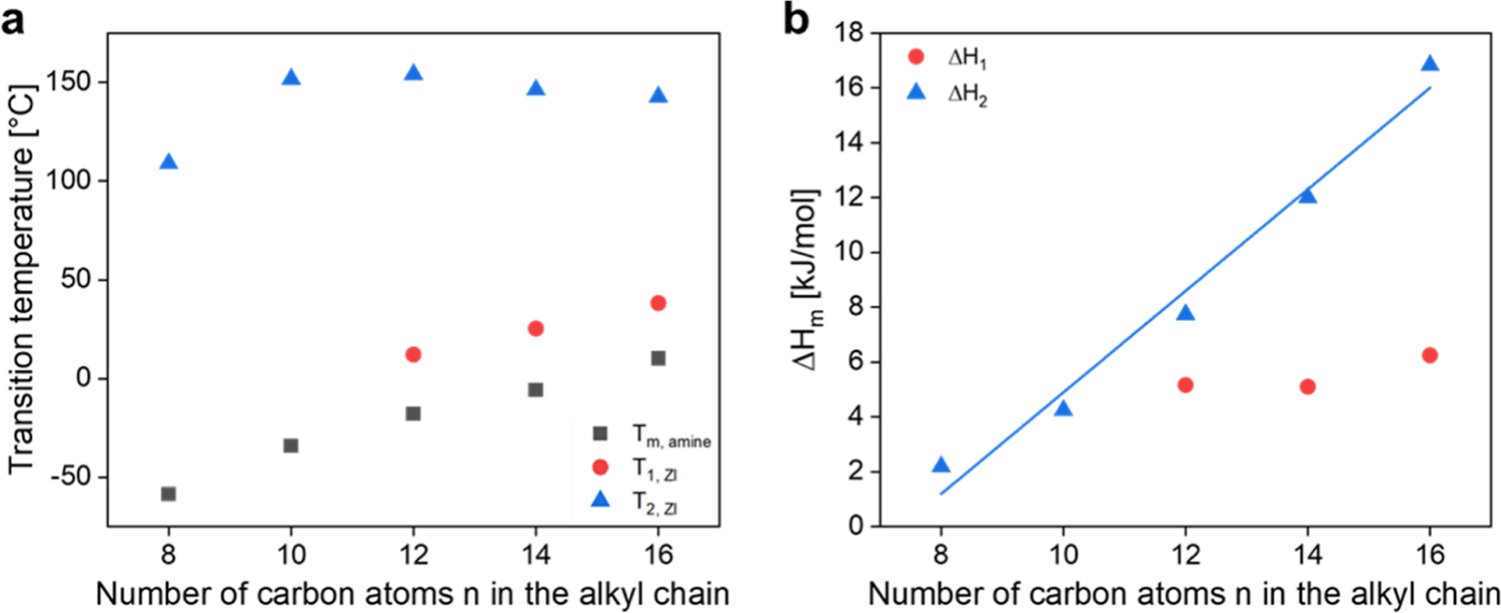
(a) Transition temperatures for ZIs and precursor amines and (b) melt enthalpies for ZIs.

Figure 5a shows the transition temperatures of the precursor amines and their respective ZIs. Interestingly, the overall trend regarding melting temperature that can be seen for the amines remains the same for *T*_1_ of the ZIs, whereas *T*_2_ does not show a linear increase with increasing chain length.

By determining the melt enthalpies Δ*H*_m_ it is possible to estimate the degree of crystallinity in the side chains. As can be seen in Figure 5b, Δ*H*_m_ at *T*_2_ (Δ*H*_2_) increases with an increasing chain length of the ZIs. However, this trend cannot be found for Δ*H*_m_ at *T*_1_ (Δ*H*_1_), as the enthalpies for this process differ only slightly and show no increase with chain length. The linearity seen for Δ*H*_2_ is represented by the following eq 1, where *z* is the number of total carbon atoms in the side chain, *k* is the contribution of each added methylene group to the overall enthalpy of the transition and the constant Δ*H*_m,e_ reflects the contribution to the enthalpy due to the chain end.^37,40^By applying this equation to the data points for *T*_2_ in Figure 5b, the value for *k* can be evaluated as (1.85 ± 0.16) kJ/mol of methylene group and Δ*H*_m,e_ as (−13.62 ± 1.92) kJ/mol.


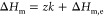
1

Assuming free rotation of the terminal methyl group, which is already approaching liquid-like behavior, its contribution can be disregarded when calculating the fraction of crystalline methylene groups. As a consequence, Δ*H*_m,e_ can be eliminated from eq 1. By using eq 2, which only accounts for the methylene groups, the number of crystalline methylene groups in the ZI terminal alkyl chain *n*_c_ can subsequently be calculated.^37,41^ The results from this calculation can be found in Table 2.
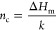
2It can clearly be seen that *n*_c_ at *T*_2_ increases with increasing chain length. Based on the *n*_c_ values at *T*_2_ it can be seen that all ZIs contain seven to eight noncrystalline methylene groups in the temperature range before *T*_2_. Moreover, when assuming the same contribution of 1.85 kJ/mol per methylene group for Δ*H*_1_, the degree of crystallinity for DmC_12_S, DmC_14_S and DmC_16_S before *T*_1_ can also be calculated. Therefore, all three ZIs contain three additional crystalline groups at temperatures below *T*_1_.

**Table 2:** Calorimetric Data for ZIs

	phase transition 1			phase transition 2		
ZI	*T*_1_ [°C]	Δ*H*_1_ [kJ/mol]^a^	*n*_c_	*T*_2_ [°C]	Δ*H*_2_ [kJ/mol]^a^	*n*_c_
DmC_16_S	37.9	6.2	3.4	142.7	16.8	9.1
DmC_14_S	27.0	5.1	2.8	145.1	12.0	6.5
DmC_12_S	11.1	5.2	2.8	154.2	7.7	4.2
DmC_10_S				151.1	4.2	2.3
DmC_8_S				109.0	2.2	1.2

a Average value taken from 2nd and 3rd heating runs.

In comparison to the transition temperature *T*_1_, the transition temperature *T*_2_ seems to follow the opposite trend, as can again be seen in Figure 5a. Here, DmC_16_S and DmC_14_S show the transition at slightly lower temperatures than DmC_12_S. In general, this ZI shows the highest *T*_2_, as this transition temperature is again lower for DmC_8_S and DmC_10_S. Therefore, the DmC_12_S has the broadest mesophase between 11–154 °C (Table 3). To further investigate and assign these phases, the analysis via polarized optical microscopy (POM) and X-ray diffraction (XRD) will be described below, as even the strongly endothermic process *T*_2_ that is visible in the DSC data of all compounds does not appear to be a conventional melting peak.

**Table 3:** Transition Temperatures for All Five ZIs^a^

	*T*_1_ [°C]	*T*_2_ [°C]	*T*_t_^DTA^ [°C]	*T*_t_^POM^ [°C]
DmC_16_S	37.9	142.7	278.9 (273.4)	269
DmC_14_S	27.0	145.1	277.9 (269.8)	272
DmC_12_S	11.1	154.2	277.0 (271.5)	267
DmC_10_S		151.1	277.7 (275.7)	268
DmC_8_S		109.0	268.6 (266.7)	262

a *T*_1_, *T*_2_: peak onset determined via DSC on 2nd heating run *T*_t_^DTA^: peak maximum determined via DTA, peak onset from DTA in brackets *T*_t_^POM^: transition point determined via POM (black image)..

All ZIs show a further phase transition T_t_^DTA^ in DTA which is very close to their decomposition temperatures T_on_ around 280 °C, Table 1. Because the decomposition is so close, this phase transition could not be investigated via DSC measurements but was qualitatively investigated via DTA and POM instead. In order to have comparable values obtained from POM and DTA we used the peak temperatures from the DTA data set and compared these data with the temperature at which the POM image turns black at the same heating rate (10 K/min).

**Figure 6:**
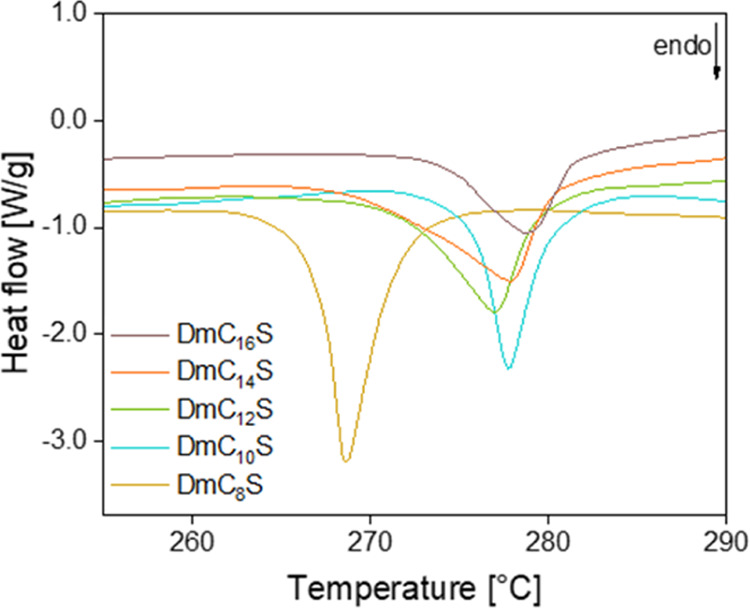
DTA traces between 255 and 290 °C.

Figure 6 shows the DTA signal that was recorded during the TGA/DTA measurements for all five ZIs in the temperature range from 255–290 °C. All DTA signals of the ZIs show a clear endothermic peak between 266 and 276 °C. We assign this signal to the transition of the ionic structures into a very low order, possibly liquid phase. This would be in agreement with clearing points for liquid crystalline systems. Indeed, these *T*_t_^DTA^ found for the ZIs are similar to clearing points found in the literature.^2,3,30,33,42,43^*T*_t_^POM^ and *T_t_*^DTA^ found for all compounds are consistent. Interestingly, the *T*_t_ of DmC_10_S to DmC_16_S are almost the same, whereas DmC_8_S shows a peak maximum that is shifted toward lower temperatures. The same behavior has already been seen for the second distinct endothermic peak *T*_2_ around 150 °C in the DSC (Figure 4), where DmC_8_S shows a phase transition at lower temperatures when compared to the longer chained ZIs. All transition temperatures can be found in Table 3. These data have been further verified using POM; these experiments are discussed below and in the Supporting Information, Figures S3–S6.

As already described, the chain length of the alkyl tail seems to have an influence on the phase transition behavior and we can therefore split the ZIs investigated in this study into two subcategories. Due to the nature of the zwitterions and the transitions seen in the DSC data, one could assume that especially the longer chained ZIs will form liquid crystals (LCs) and that the states observed between 10–150 °C and above 150 °C to *T*_t_^DTA^ might be typical LC phases.

Indeed, liquid crystalline ZIs have already been reported. For example, Soberats et al. investigated wedge-shaped imidazolium-type zwitterions that contained an alkoxybenzene unit with three C_12_ alkyl chains.^33^ The anion moiety of the zwitterions consisted of a sulfonate or a dicyanoethanolate which were tethered by a propyl group. These ZIs form columnar hexagonal (Col_h_) phases from room temperature to around 200 °C. ZIs with an imidazolium moiety and different alkyl chain lengths were studied by Rondla et al. These authors also used the sulfonate group as the negatively charged fragment in their compounds and changed the alkyl chains from C_12_ to C_18_. These ZIs showed LC phase behavior and decomposition between 220–250 °C. C_14_, C_16_ and C_18_ additionally showed a soft phase before transitioning into a liquid crystal.^43^

Lin et al. also investigated the imidazolium moiety as a starting point for their ZIs. The authors also changed the chain length of the alkyl chain attached to the imidazolium from C_10_ to C_18_. The anion moiety for their ZIs was given by a carboxylate attached to the second N atom of the imidazolium ring. For all of their ZIs the authors reported two crystalline phases followed by a SmA phase. The transition between Cr_2_ and SmA appears around 110 °C for C_12_ to C_18_, whereas the transition can be seen at 39 °C for the C_10_ compound with a comparably low transition enthalpy. This ZI also shows reversible heating/cooling behavior in contrast to the longer chained compounds starting with C_12_, as these all show decomposition between 190–230 °C.^42^

**Figure 7:**
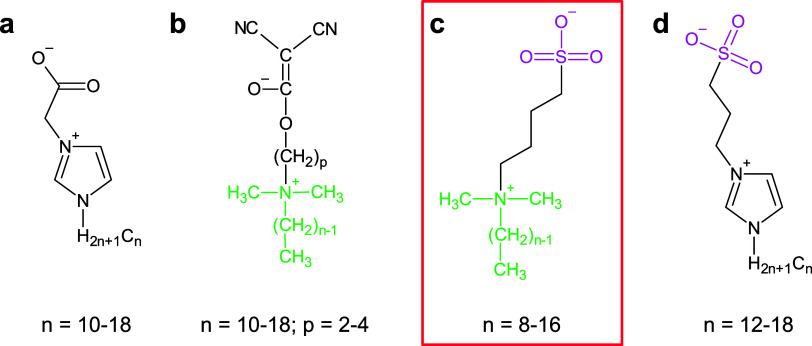
ZIs found in the literature: (a) work by Lin et al.,^42^ (b) work by Mathis et al.,^30^ (c) ZIs presented in this study, and (d) work by Rondla et al.^43^

Mathis et al. reported on the same dimethylalkylammonium cation moieties that were used in this study in their ZIs with dicyanoethenolates as anionic unit. In their investigations they also looked at the influence of the spacer length between cation and anion moiety and found different LC phases for various spacer and alkyl chain lengths. Their 16–2 ZI (C_16_ alkyl chain and C_2_ spacer), for example, showed a SmA phase before decomposition at 200 °C. The 16–3 ZI exhibited cubic and SmA phases and the 16–4 ZI only a Col_h_ phase. For the C_18_ ZIs the authors reported SmA (and cubic) phases with decompositions around 240 °C.^30^ Structural comparisons between the ZIs investigated in this study and selected LCs reported in the literature can be seen in Figure 7.

**Figure 8:**
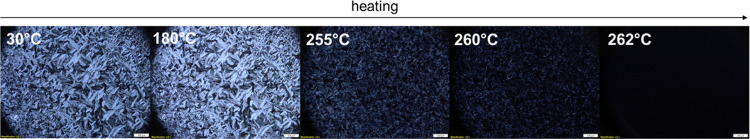
POM images of DmC_8_S up to *T*_t_.

Based on these reports it is quite feasible that the ZIs investigated in this study should also show LC phases. Somewhat surprisingly, polarized optical microscopy (POM) does not show all of the phase transitions that are so clearly visible for all compounds in the DSC (Supporting Information, Figures S3–S6). In most cases the transitions–if at all–only appear as slight changes in brightness or coloration. Only DmC_8_S and DmC_10_S also show a slight structural change at higher temperatures. As discussed before, the POM images can, however, help to determine the temperature of the latest phase transition just before ZI decomposition, Figure 8. As stated above in the discussion of the DTA data the *T*_t_ determined via POM correspond nicely with those found in the DTA scans. Qualitatively, this is supported by the fact that in POM, all textures that were previously visible, disappear at the determined *T*_t_^POM^, as seen in Figure 8 for DmC_8_S (black image at 262 °C).

**Figure 9:**
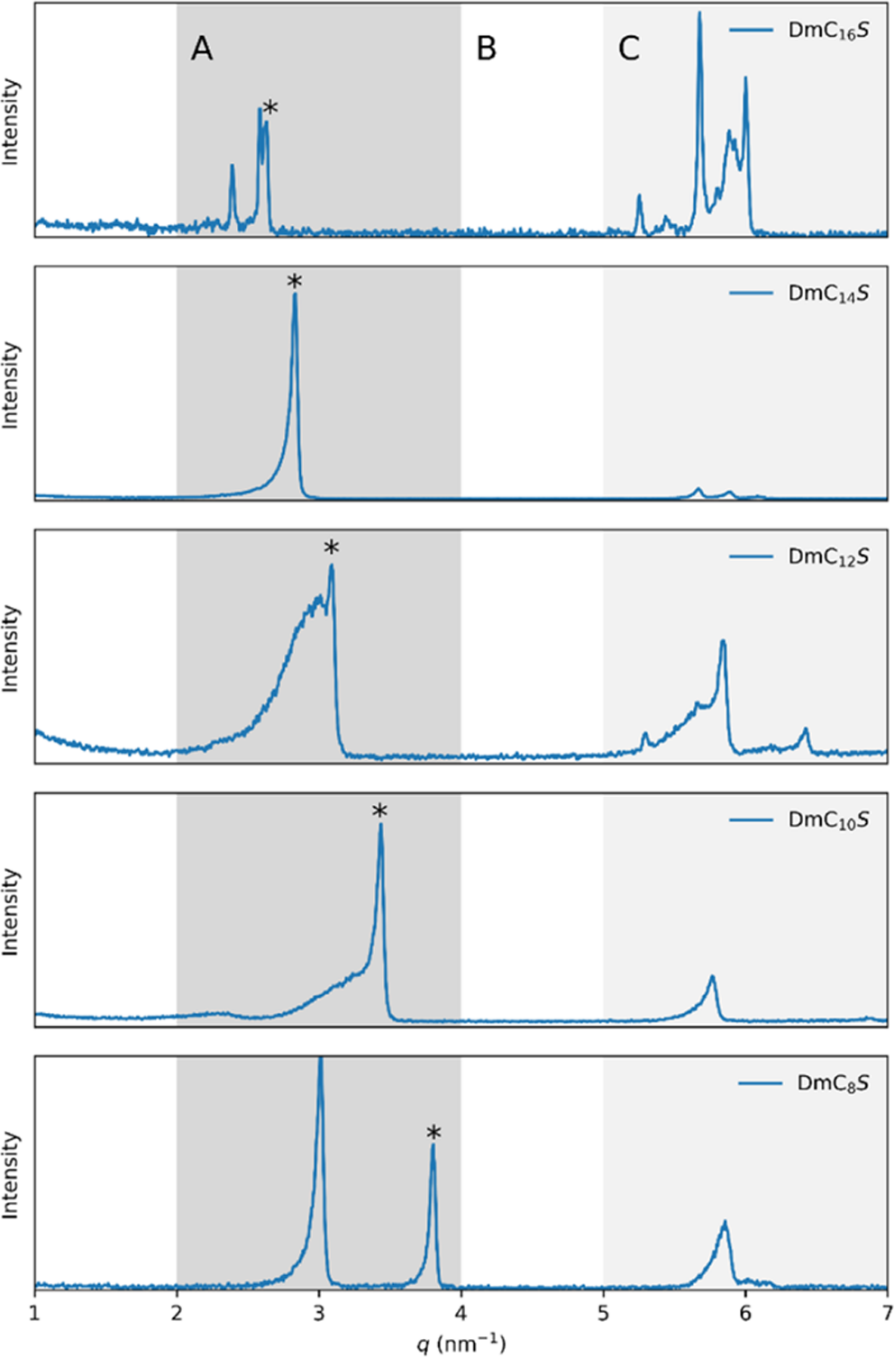
Overview of SAXS data of the ZIs measured at a temperature of 20 °C. Marked areas are **A** (2 nm^–1^ < *q* < 4 nm^–1^), **B** (4 nm^–1^ < *q* < 5 nm^–1^) and **C** (5 nm^–1^ < *q* < 7 nm^–1^).

Small-angle X-ray scattering analysis was selected to further investigate the mesoscopic structure of the ZIs due to the inability of POM images to explicitly assign phases. SAXS experiments were conducted over a temperature range of 20 to 284 °C (temperature calibration see Figure S7). An overview of the resulting data measured at 20 °C is depicted in Figure 9. The curves of all samples show multiple reflections in the *q*-range of 1 to 7 nm^–1^. No systematic change in the peak pattern is apparent with varying alkyl chain lengths. However, the peaks can be divided into two separate regions.

The first set of peaks is located in the *q*-range of 2 to 4 nm^–1^, while the second is found between 5 and 7 nm^–1^. These regions are labeled as A and C in Figure 9. None of the samples exhibit peaks between 4 and 5 nm^–1^ (labeled as B). This general trend persists at higher temperatures, as shown in Figures S8 to S12. There are distinct peaks in the SAXS curves of all samples at temperatures ranging from 20 to 275 °C. Therefore, we can conclude that all samples exhibit mesomorphic ordered structures from 20 °C up to at least 275 °C. The peaks disappeared in the SAXS patterns of DmC_8_S, DmC_14_S, and DmC_16_S at 284 °C. For DmC_10_S and DmC_12_S, the peaks began to broaden at the same temperature. The data suggests that all samples transition to an amorphous state at temperatures of approximately 284 °C. After the SAXS measurements, the samples turned brownish black, indicating decomposition around the clearing transition, confirming the TGA results.

For interpretation of the SAXS pattern, one can assume a nanosegregation of the ionic zwitterionic groups and the alkyl tails for thermodynamic reasons. Tentatively, we propose the existence of a layered nanostructure for the ZIs. This structure features a separation of alkyl chains from the zwitterionic groups, resulting in alkyl-chain-rich and zwitterionic-rich layers. This assumption is supported by a recent study conducted by Gustavsson et al., who revealed similar lamellar structures for the zwitterion bis-*n*-tetradecylphosphobetaine.^39^ Their study shows a highly complex SAXS pattern due to the coexistence of multiple lamellar phases. In analogy, it is likely that the complex peak pattern in the curves of DmC_12_S and DmC_16_S could be explained similarly.

**Figure 10:**
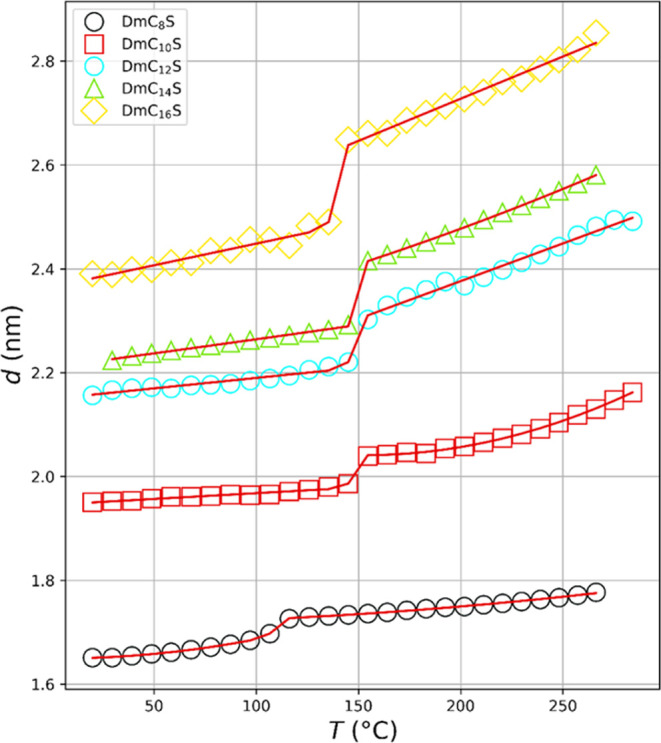
Long periods of the lamellar structures as a function of temperature (symbols) and fit curves applying eq 3 (red solid lines).

For simplicity, we initially concentrate on the peak identified at the highest *q*-value in region A for each sample. This peak is denoted by an asterisk in Figure 9. We assume that this represents the interlayer spacing of a lamellar mesophase, corresponding to the (001) peak of a lamellar phase. Under this assumption, the long periods of the lamellar structures were calculated from the maximum peak positions as *d* = 2π\/*q*_max_. The resulting *d*-values increase with longer alkyl chain length of the ZI and rise with higher temperature, as shown in Figure 10. At a temperature as low as 20 °C, the *d*-values increase as follows: 1.65 nm (DmC_8_S), 1.95 nm (DmC_10_S), 2.16 nm (DmC_12_S), 2.22 nm (DmC_14_S), 2.39 nm (DmC_16_S). Maximum values are *d* = 1.78 nm (DmC_8_S), 2.16 nm (DmC_10_S), 2.49 nm (DmC_12_S), 2.58 nm (DmC_14_S) and 2.85 nm (DmC_16_S). Overall, these values correspond to a temperature-induced increase in the long period of 7% (DmC_8_S), 10% (DmC_10_S), 13% (DmC_12_S), 14% (DmC_14_S), and 16% (DmC_16_S). A significant characteristic of all curves is the stepwise increase in the long period at approximately 110 °C for DmC_8_S and around 150 °C for the other samples. It is also noticeable that the step height increases by 0.03 nm (DmC_8_S), 0.05 nm (DmC_10_S), 0.08 nm (DmC_12_S), 0.12 nm (DmC_14_S) and 0.16 nm (DmC_16_S).

The position and height of this step correlate with the second thermal transition found in the DSC. It has already been shown that the heat enthalpies of the second transition increase as the alkyl chain length increases (Figure 5b). For samples DmC_10_S to DmC_16_S the transition temperatures are in the range of *T*_2_ = 142 to 154 °C. By contrast, DmC_8_S has a significantly lower transition temperature of *T*_2_ = 109 °C. It appears that DmC_8_S has different thermodynamic properties compared to the other ZIs. Both DSC and SAXS data are indicative for a first-order phase transition at *T*_2_. Next, we will provide a more detailed characterization of the thermal behavior of the ZIs.

The thermal expansion of the lamellar long period *d*(*T*) is characterized by a monotonic increase at temperatures below the phase transition, a stepwise increase at the transition, and a monotonic increase above the transition. This behavior can be quantified by
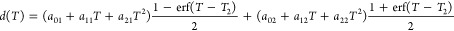
3Here, the second-order polynomials *a*_01_ + *a*_11_*T* + *a*_21_*T*^2^ and *a*_02_ + *a*_12_*T* + *a*_22_*T*^2^ represent the change of *d* at temperatures below and above the transition temperature *T*_2_. The error function erf models the stepwise increase at the transition temperature *T*_2_. Applying the equation above for curve fitting the *d*-values describes them well over the entire temperature range, as can be seen in Figure 10 (red solid lines). While a linear approximation is usually sufficient to measure material expansion, higher-order polynomials are also utilized. For example, the thermal expansion of copper can be modeled using a third-order polynomial equation (for details see James et al.^44^).

**Figure 11:**
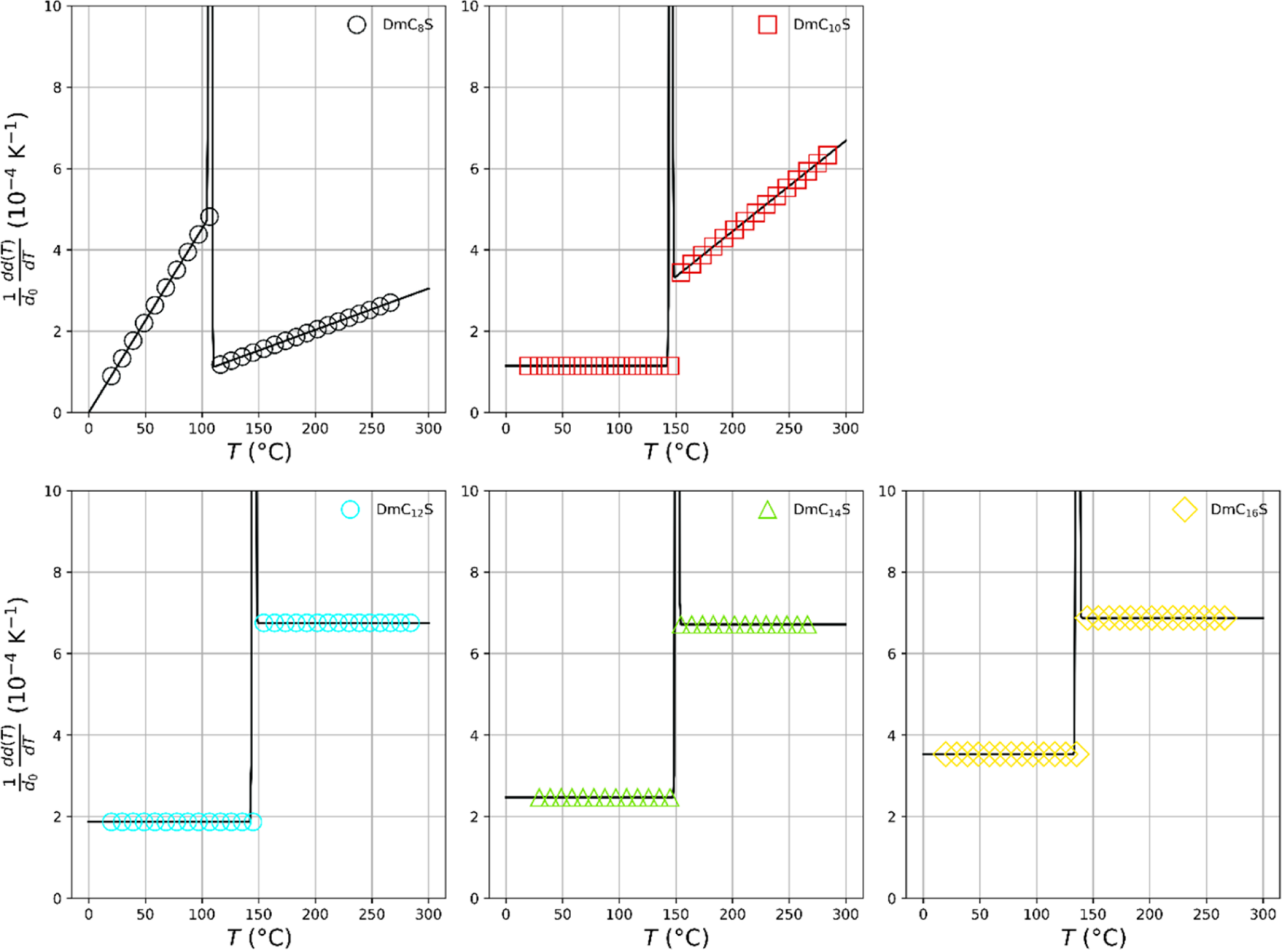
Thermal expansion of the long periods of the lamellar structures as a function of temperature according to eq 4 (black solid lines) and according to the approximation given in eq 5 (symbols).

Useful for further characterization of the thermal expansion is the derivative of *d*(*T*) to *T*, which after normalization to the initial value of *d*, *d*_0_, is
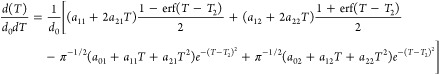
4When the system is far enough from the phase transition temperature *T*_2_, this expression can be approximated by
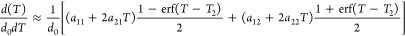
5which provides (*a*_11_ + 2*a*_21_*T*)/*d*_0_ for temperatures lower than *T*_2_ and (*a*_12_ + 2*a*_22_*T*)/*d*_0_ for temperatures higher than *T*_2_. The course of the -values according to eq 4 and according to the approximation eq 5 are shown in Figure 11 (symbols and black solid lines, respectively). Two distinct types of expansion are apparent. The  is either constant or increases linearly with temperature. The first case refers to *a*_1*i*_/*d*_0_ = α_*i*_, which represents the classical isobaric uniaxial thermal expansion coefficient. The change in the α_*i*_ along a certain direction with temperature in the linear approximation is typically expressed in 10^–6^ K^–1^.^45^ The thermal expansion of DmC_8_S is quadratic in temperature with values of *a*_21_ = 3.7 × 10^–6^ nm K^–2^ below *T*_2_ = 107 °C and *a*_22_ = 8.4 × 10^–6^ nm K^–2^ above *T*_2_. We see another quadratic expansion only for DmC_10_S above *T*_2_ with *a*_22_ = 2.2 × 10^–6^ nm K^–2^. Below *T*_2_, a linear behavior with increasing expansion coefficient is found for all samples, except for DmC_8_S, in the line a_1_ = 1.1 × 10^–4^ K^–1^ (DmC_10_S), 1.9 × 10^–4^ K^–1^ (DmC_12_S), 2.5 × 10^–4^ K^–1^ (DmC_14_S) and 3.5 × 10^–4^ K^–1^ (DmC_16_S). Above *T*_2_, high a_1_-values of 6.7 × 10^–4^ K^–1^ (DmC_12_S), 6.7 × 10^–4^ K^–1^ (DmC_14_S) and 6.9 × 10^–4^ K^–1^ (DmC_16_S), were determined (Table 4). In literature, Watanabe et al. report the thermal expansion of layer structures in smectic liquid crystals.^46^ These authors reported *a*-values of smectic layers of a PEO_114_-*b*-PMA(Az)_45_ block copolymer between 3.8 × 10^–4^ and 7.2 × 10^–3^ K^–1^ at different temperatures which is a surprisingly large range of values. For comparison, the mean uniaxial thermal expansion coefficient is 0.714 × 10^–4^ K^–1^ for some 745 different crystalline organic compounds.^45^ Interestingly, 34% of these structures may have at least one orthogonal axis with negative thermal expansion.

**Table 4:** Thermal Expansion Characteristics of the Lamellar Long Period, *d*. *T*_2_: Transition Temperature; Δ*d*: Height of the Stepwise Increase of the Long Period at *T*_2_; *a*_01_, *a*_11_, *a*_21:_ Coefficients of the Thermal Expansion Below *T*_2_; *a*_02_, *a*_12_, *a*_22_: Coefficients of the Thermal Expansion Above *T*_2_; *a*_1_, *a*_2_: Linear Expansion Coefficients Below and Above *T*_2_, Respectively

	*T*_2_ [°C]	Δ*d* [nm]	*a*_01_ [nm]	*a*_11_ [10^–4^ nm K^–1^]	*a*_21_ [10^–6^ nm K^–2^]	*a*_02_ [nm]	*a*_12_ [10^–4^ nm K^–1^]	*a*_22_ [10^–6^ nm K^–2^]	*a*_1_ [10^–4^ K^–1^]	*a*_2_ [10^–4^ K^–1^]
DmC_16_S	136	0.16	2.37	8.4		2.40	16.2		3.5	6.9
DmC_14_S	151	0.12	2.21	5.5		2.18	14.8		2.5	6.7
DmC_12_S	146	0.08	2.15	4.0		2.09	14.5		1.9	6.7
DmC_10_S	146	0.05	1.95	2.2		1.98		2.2	1.1	
DmC_8_S	107	0.03	1.65		3.7	1.72		8.4		

With increasing temperatures, the thermal expansion coefficients of polyethylene fibers in the *a* and *b* directions were reported to be approximately 2.7 × 10^–4^ and 6.1 × 10^–6^ K^–1^, respectively, by Hsieh and Hu.^47^ The much larger thermal expansion coefficient along the *a*-direction explains the ease of transformation from the orthorhombic crystals form to the pseudohexagonal form upon heating.

A closer understanding of the peak patterns is needed to learn more about the ZIs’ structures. The DmC_14_S appears to have the simplest peak pattern as a function of temperature (see Figure S11). We will therefore consider DmC_14_S next.

**Figure 12:**
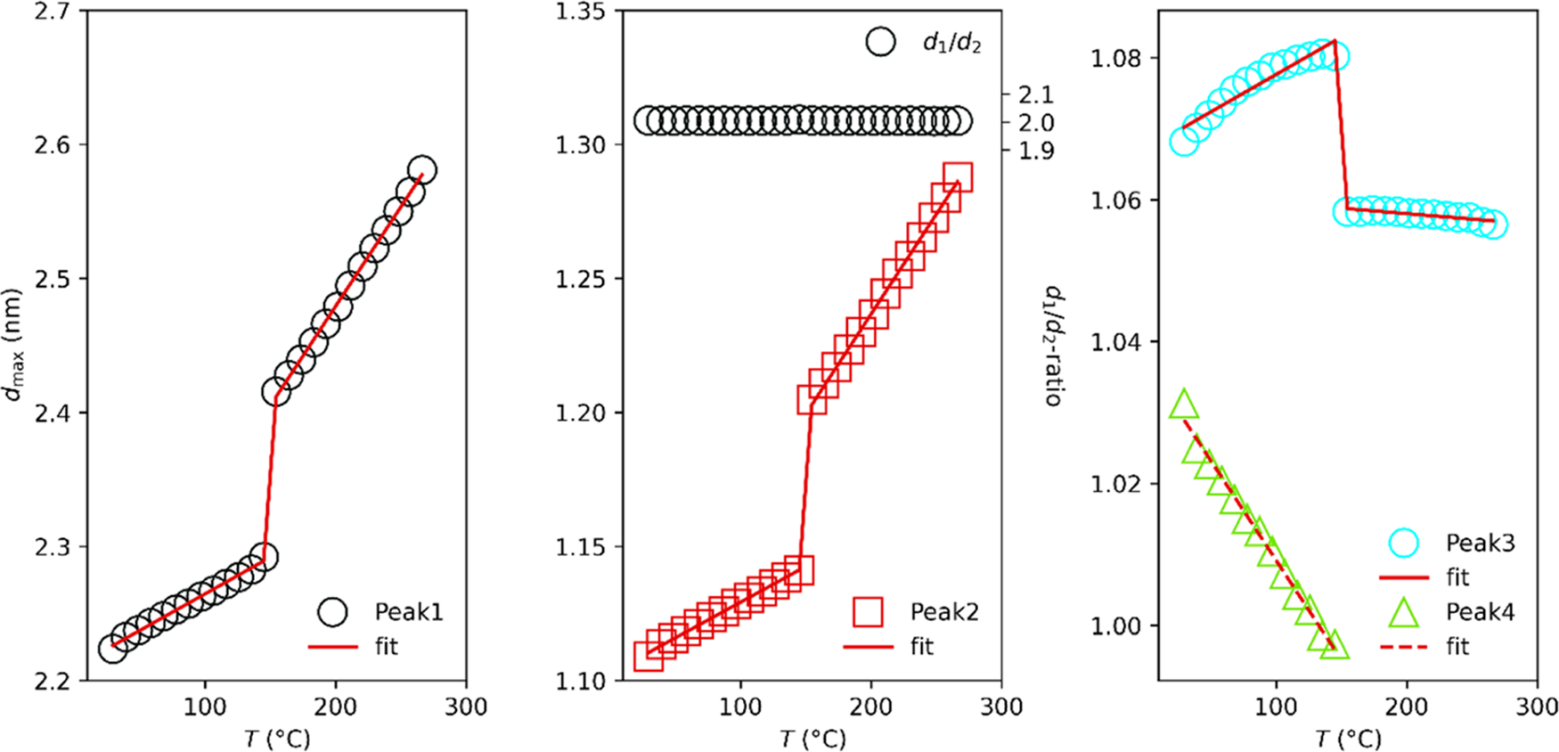
Changes in the *d*-values of the reflection of DmC_14_S with temperature are represented by symbols (curve fits by lines). On the left and middle, the *d*-values of the first and second peaks exhibit similar characteristics. The ratio *d*_1_/*d*_2_ remains constant at two across all temperatures. On the right, the changes in the *d*-values of the third and fourth peaks are illustrated.

First, we approximate visible peaks found in the data with Lorentzian peak profiles, as shown for DmC_14_S in Figure S13. Four peaks are found with maxima at *q*_1_ = 2.21 nm^–1^, *q*_2_ = 1.10 nm^–1^, *q*_3_ = 1.06 nm^–1^ and *q*_4_ = 1.04 nm^–1^. If we assign *q*_1_ to the (001) reflection and *q*_2_ to the (002) reflection, we obtain a long period for the lamella of π/*q*_2_ = 2.84 nm. Changes in the *d*-values of the reflection of DmC_14_S with temperature are represented by symbols (curve fits by lines) in Figure 12. On the left and middle, the *d*-values of the first and second peaks exhibit similar characteristics. The ratio *d*_1_/*d*_2_ remains constant at two across all temperatures. On the right, the changes in the *d*-values of the third and fourth peaks are illustrated.

**Scheme 1:**
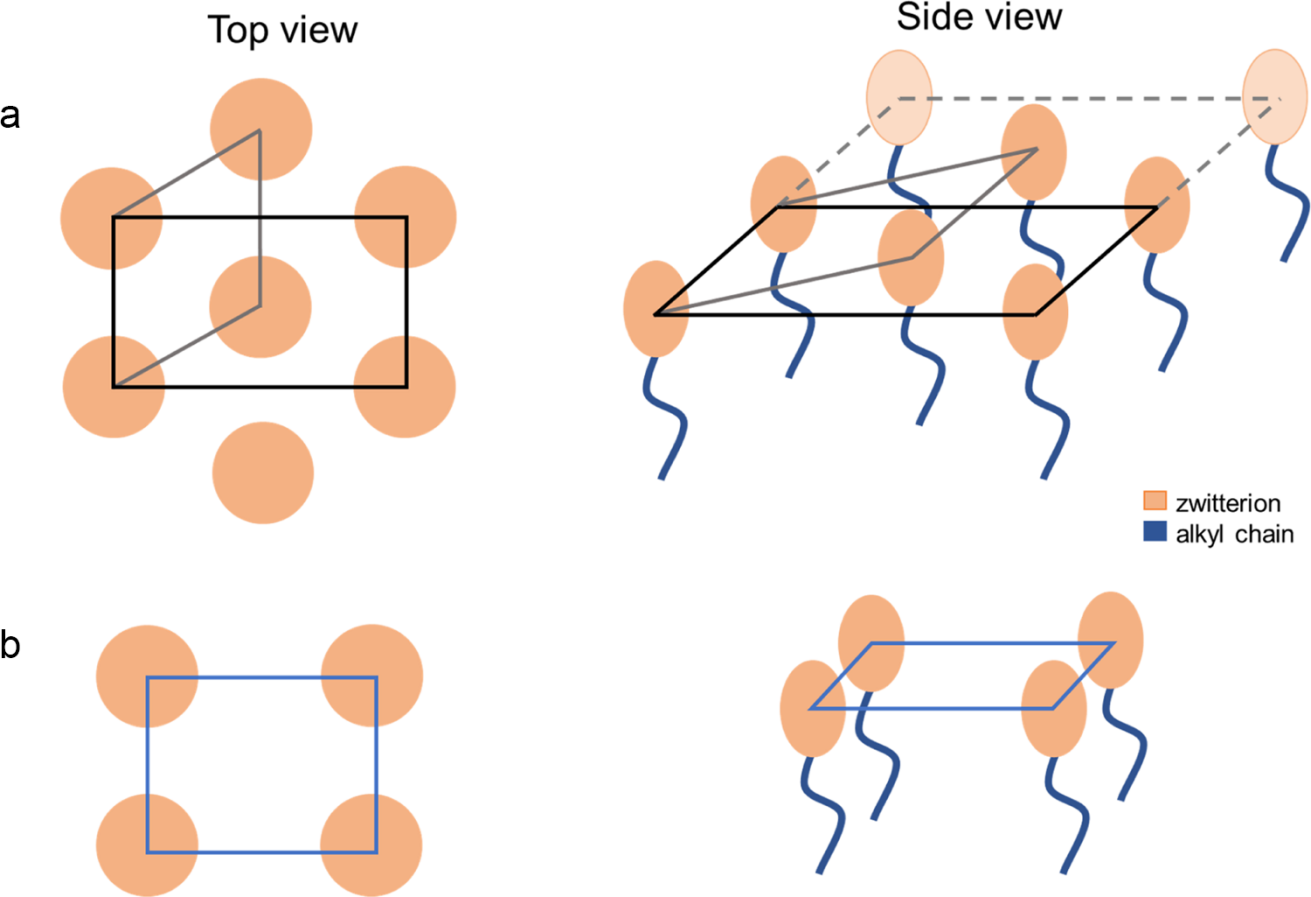
Graphical Representation of Possible Molecular Arrangement in the Unit Cells: (a) Hexagonal and (b) Orthorhombic

Tentatively, we assume a primitive orthorhombic two-dimensional lattice for the in-plane structure at temperatures below the phase transition *T*_2_ and a two-dimensional hexagonal structure above. The ordering within the plane is probably due to the arrangement of the zwitterionic head groups. A possible arrangement of the ZIs in both unit cells is shown in Scheme 1.

**Figure 13:**
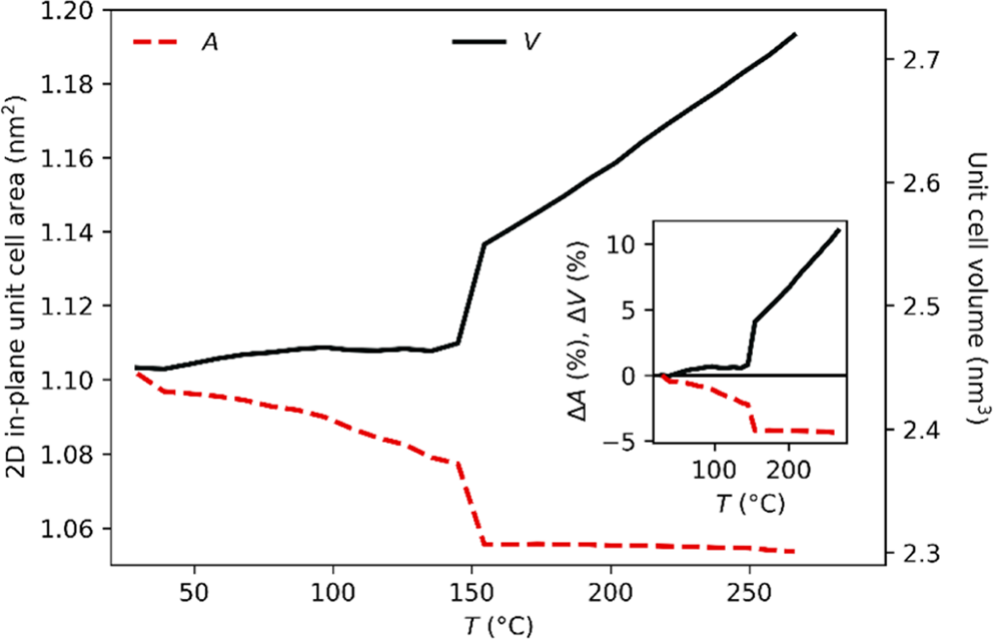
Changes in the in-plane area *A* (in nm^2^) and volume of the unit cell *V* (in nm^3^) for DmC_14_S (red dashed and black solid lines, respectively). Inset. Changes of *A* and *V* (both in %) with respect to a temperature of 21 °C.

The first two reflections of the two-dimensional orthorhombic lattice are indexed as (100) and (010). The lattice constants are given by *a*_*o*_ = *d*_100_ and *b*_*o*_ = *d*_010_ and the in-plane area of the lattice cell is *A*_*o*_ = *a*_*o*_*b*_*o*_. The lattice constant of the two-dimensional hexagonal lattice is calculated from the (100) reflection as , and with an area of . The results are shown in Figure 13. The in-plane area decreases from 1.12 to 1.054 nm^2^, which corresponds to a decrease of 4.6%. Surprisingly, the two-dimensional cell is shrinking. This is probably due to a continuous reorganization of the zwitterionic groups with increasing temperature. In any case, we expect the entire unit cell to expand, usually with the temperature. The temperature-dependent volume of the unit cell, calculated as the product of the two-dimensional unit cell with the lamellar long period, is shown in Figure 13. The volume increases from 2.449 to 2.710 nm^3^, corresponding to an increase of 10%. This means that DmC_14_S expands overall but is anisotropic in different directions.

Currently, we cannot suggest in-plane structures for the other ZIs of this study since their scattering patterns are ambiguous. This is probably due to polymorphic lamellar structures, as reported by Gustavsson et al. for phosphobetaine based ZIs.^39^ Also, similarly, triglycerides display highly polymorphic phases and phase transitions.^48^

As a result of the X-ray, POM and DSC data it is very difficult to clearly assign either soft-solid (plastic crystal) or true liquid crystalline phases. During the POM experiments we have noticed however, that the phase observed at lower temperatures (below the intense *T*_2_ signal in the DSC) appears much harder than the phase above *T*_2_, when pushing on the samples with a spatula. This is a qualitative observation, but it may indicate that the first phase below *T*_2_ might be a soft solid while the phase above *T*_2_ may possibly be a liquid crystalline phase. While we cannot rule out other possibilities such a behavior would be consistent with previous observations.^43^

## Conclusions

Sulfobetaine zwitterions made from *n*-alkyl dimethylamines and butanesultone are easily synthesized in multigram amounts; the synthesis is scalable and provides access to liquid crystalline ZIs with rather high thermal stabilities up to over 200 °C with lamellar LC phases. The fact that the LC window can be tuned simply by the alkyl chain length makes them interesting for a series of applications such as ion transporting membranes. Moreover, the LC windows are rather large and in a temperature window that is indeed interesting for applications such as batteries or intermediate temperature fuel cells. As such, these zwitterions are candidates for the development of next generation advanced membranes for e.g., energy applications.

## Supporting Information Available

The Supporting Information file contains additional POM, DSC and SAXS data, as well as the ESI-MS and ^1^H NMR spectra for the ZIs (PDF)

## References

[ref1] Ichikawa T. Zwitterions as building blocks for functional liquid crystals and block copolymers. Polym. J. 2017, 49 (5), 413–421. 10.1038/pj.2017.6.

[ref2] Narita A.; Shibayama W.; Ohno H. Structural factors to improve physico-chemical properties of zwitterions as ion conductive matrices. J. Mater. Chem. 2006, 16 (15), 1475 10.1039/b515287a.

[ref3] Ohno H. Functional Design of Ionic Liquids. Bull. Chem. Soc. Japan 2006, 79 (11), 1665–1680. 10.1246/bcsj.79.1665.

[ref4] Ohno H.; Yoshizawa-Fujita M.; Kohno Y. Design and properties of functional zwitterions derived from ionic liquids. Phys. Chem. Chem. Phys. 2018, 20 (16), 10978–10991. 10.1039/C7CP08592C. 29620779

[ref5] Yoshizawa-Fujita M.; Ohno H. Applications of Zwitterions and Zwitterionic Polymers for Li-Ion Batteries. Chem. Record 2023, 23, e202200287 10.1002/tcr.202200287. 36782072

[ref6] Chattopadhyay A.; Harikumar K. G. Dependence of critical micelle concentration of a zwitterionic detergent on ionic strength: implications in receptor solubilization. FEBS Lett. 1996, 391 (1–2), 199–202. 10.1016/0014-5793(96)00733-8. 8706916

[ref7] Palladino P.; Rossi F.; Ragone R. Effective critical micellar concentration of a zwitterionic detergent: a fluorimetric study on n-dodecyl phosphocholine. J. Fluoresc. 2010, 20 (1), 191–196. 10.1007/s10895-009-0537-0. 19756982

[ref8] Shiloach A.; Blankschtein D. Prediction of Critical Micelle Concentrations and Synergism of Binary Surfactant Mixtures Containing Zwitterionic Surfactants. Langmuir 1997, 13 (15), 3968–3981. 10.1021/la970160x.

[ref9] Shen H.; Zhou M.; Zhang Q.; Keller A.; Shen Y. Zwitterionic light-responsive polymeric micelles for controlled drug delivery. Colloid Polym. Sci. 2015, 293 (6), 1685–1694. 10.1007/s00396-015-3550-7.

[ref10] Harijan M.; Singh M. Zwitterionic polymers in drug delivery: A review. Journal of molecular recognition: JMR 2022, 35 (1), e2944 10.1002/jmr.2944. 34738272

[ref11] Chen S.-H.; Chang Y.; Lee K.-R.; Wei T.-C.; Higuchi A.; Ho F.-M.; Tsou C.-C.; Ho H.-T.; Lai J.-Y. Hemocompatible control of sulfobetaine-grafted polypropylene fibrous membranes in human whole blood via plasma-induced surface zwitterionization. Langmuir 2012, 28 (51), 17733–17742. 10.1021/la3036902. 23181727

[ref12] Kuo W.-H.; Wang M.-J.; Chien H.-W.; Wei T.-C.; Lee C.; Tsai W.-B. Surface modification with poly(sulfobetaine methacrylate-co-acrylic acid) to reduce fibrinogen adsorption, platelet adhesion, and plasma coagulation. Biomacromolecules 2011, 12 (12), 4348–4356. 10.1021/bm2013185. 22077421

[ref13] Baggerman J.; Smulders M. M. J.; Zuilhof H. Romantic Surfaces: A Systematic Overview of Stable, Biospecific, and Antifouling Zwitterionic Surfaces. Langmuir 2019, 35 (5), 1072–1084. 10.1021/acs.langmuir.8b03360. 30620199 PMC6365910

[ref14] Cheng Q.; Asha A. B.; Liu Y.; Peng Y.-Y.; Diaz-Dussan D.; Shi Z.; Cui Z.; Narain R. Antifouling and Antibacterial Polymer-Coated Surfaces Based on the Combined Effect of Zwitterions and the Natural Borneol. ACS Appl. Mater. Interfaces 2021, 13 (7), 9006–9014. 10.1021/acsami.0c22658. 33576614

[ref15] van Andel E.; Lange S. C.; Pujari S. P.; Tijhaar E. J.; Smulders M. M. J.; Savelkoul H. F. J.; Zuilhof H. Systematic Comparison of Zwitterionic and Non-Zwitterionic Antifouling Polymer Brushes on a Bead-Based Platform. Langmuir 2019, 35 (5), 1181–1191. 10.1021/acs.langmuir.8b01832. 30265555 PMC6366122

[ref16] Wu A.; Gao Y.; Zheng L. Zwitterionic amphiphiles: their aggregation behavior and applications. Green Chem. 2019, 21 (16), 4290–4312. 10.1039/C9GC01808E.

[ref17] Shahkaramipour N.; Jafari A.; Tran T.; Stafford C. M.; Cheng C.; Lin H. Maximizing the grafting of zwitterions onto the surface of ultrafiltration membranes to improve antifouling properties. J. Membr. Sci. 2020, 601, 117909 10.1016/j.memsci.2020.117909. PMC7539668 33041468

[ref18] Ventura C.; Guerin A. J.; El-Zubir O.; Ruiz-Sanchez A. J.; Dixon L. I.; Reynolds K. J.; Dale M. L.; Ferguson J.; Houlton A.; Horrocks B. R.; Clare A. S.; Fulton D. A. Marine antifouling performance of polymer coatings incorporating zwitterions. Biofouling 2017, 33 (10), 892–903. 10.1080/08927014.2017.1383983. 29083230

[ref19] Zhang Y.; Liu Y.; Ren B.; Zhang D.; Xie S.; Chang Y.; Yang J.; Wu J.; Xu L.; Zheng J. Fundamentals and applications of zwitterionic antifouling polymers. J. Phys. D: Appl. Phys. 2019, 52 (40), 403001 10.1088/1361-6463/ab2cbc.

[ref20] Shahkaramipour N.; Ramanan S. N.; Fister D.; Park E.; Venna S. R.; Sun H.; Cheng C.; Lin H. Facile Grafting of Zwitterions onto the Membrane Surface To Enhance Antifouling Properties for Wastewater Reuse. Ind. Eng. Chem. Res. 2017, 56 (32), 9202–9212. 10.1021/acs.iecr.7b02378.

[ref21] Kobayashi T.; Ichikawa T.; Kato T.; Ohno H. Development of Glassy Bicontinuous Cubic Liquid Crystals for Solid Proton-Conductive Materials Adv. Mater. 2017; Vol. 29 (4), 10.1002/adma.201604429. 27882615

[ref22] Zehbe K.; Lange A.; Taubert A. Stereolithography Provides Access to 3D Printed Ionogels with High Ionic Conductivity. Energy Fuels 2019, 33 (12), 12885–12893. 10.1021/acs.energyfuels.9b03379.

[ref23] Delahaye E.; Göbel R.; Löbbicke R.; Guillot R.; Sieber C.; Taubert A. Silica ionogels for proton transport. J. Mater. Chem. 2012, 22 (33), 17140 10.1039/c2jm00037g.

[ref24] Taubert A.; Löbbicke R.; Kirchner B.; Leroux F. First examples of organosilica-based ionogels: synthesis and electrochemical behavior. Beilstein J. Nanotechnol. 2017, 8, 736–751. 10.3762/bjnano.8.77. 28487817 PMC5389198

[ref25] Wojnarowska Z.; Lange A.; Taubert A.; Paluch M. Ion and Proton Transport In Aqueous/Nonaqueous Acidic Ionic Liquids for Fuel-Cell Applications-Insight from High-Pressure Dielectric Studies. ACS Appl. Mater. Interfaces 2021, 13 (26), 30614–30624. 10.1021/acsami.1c06260. 34164974 PMC8289238

[ref26] Behera K.; Pandey S. Interaction between ionic liquid and zwitterionic surfactant: a comparative study of two ionic liquids with different anions. J. Colloid Interface Sci. 2009, 331 (1), 196–205. 10.1016/j.jcis.2008.11.008. 19027123

[ref27] Cheng C.; Qu G.; Wei J.; Yu T.; Ding W. Thermodynamics of Micellization of Sulfobetaine Surfactants in Aqueous Solution. J. Surfact Detergents 2012, 15 (6), 757–763. 10.1007/s11743-012-1374-8.

[ref28] Wieczorek D.; Gwiazdowska D.; Staszak K.; Chen Y.-L.; Shen T.-L. Surface and Antimicrobial Activity of Sulfobetaines. J. Surfact Detergents 2016, 19 (4), 813–822. 10.1007/s11743-016-1838-3.

[ref29] Okada T. Approaches to separation interfaces. Anal. Chim. Acta 2005, 540 (1), 139–145. 10.1016/j.aca.2004.10.051.

[ref30] Mathis A.; Galin M.; Galin J. C.; Heinrich B.; Bazuin C. G. Long alkyl chain dimethylammonioalkoxydicyanoethenolates as new zwitterionic thermotropic liquid crystals. Liq. Cryst. 1999, 26 (7), 973–984. 10.1080/026782999204327.

[ref31] Infrared and Raman Spectroscopy; Elsevier, 2018 10.1016/C2015-0-00806-1.

[ref32] Yoshizawa-Fujita M.; Tamura T.; Takeoka Y.; Rikukawa M. Low-melting zwitterion: effect of oxyethylene units on thermal properties and conductivity. Chem. Commun. 2011, 47 (8), 2345–2347. 10.1039/C0CC03754K. 21170427

[ref33] Soberats B.; Yoshio M.; Ichikawa T.; Ohno H.; Kato T. Zwitterionic liquid crystals as 1D and 3D lithium ion transport media. J. Mater. Chem. A 2015, 3 (21), 11232–11238. 10.1039/C5TA00814J.

[ref34] Hemp S. T.; Zhang M.; Allen M. H.; Cheng S.; Moore R. B.; Long T. E. Comparing Ammonium and Phosphonium Polymerized Ionic Liquids: Thermal Analysis, Conductivity, and Morphology. Macro Chem. Phys. 2013, 214 (18), 2099–2107. 10.1002/macp.201300322.

[ref35] Bureš F. Quaternary Ammonium Compounds: Simple in Structure, Complex in Application. Top. Current Chem. 2019, 377 (3), 14 10.1007/s41061-019-0239-2. 31062103

[ref36] Watanabe J.; Ono H.; Uematsu I.; Abe A. Thermotropic polypeptides. 2. Molecular packing and thermotropic behavior of poly(L-glutamates) with long n-alkyl side chains. Macromolecules 1985, 18 (11), 2141–2148. 10.1021/ma00153a013.

[ref37] Thünemann A. F.; General S. Poly(ethylene imine) n-Alkyl Carboxylate Complexes. Langmuir 2000, 16 (24), 9634–9638. 10.1021/la000991u.

[ref38] Ponomarenko E. A.; Tirrell D. A.; MacKnight W. J. Water-Insoluble Complexes of Poly(l -Lysine) with Mixed Alkyl Sulfates: Composition-Controlled Solid State Structures. Macromolecules 1998, 31 (5), 1584–1589. 10.1021/ma971388u.

[ref39] Gustavsson L.; Lv Z.-P.; Cherian T.; Seppälä W.; Liljeström V.; Peng B.; Huotari S.; Rannou P.; Ikkala O. Heating-Induced Switching to Hierarchical Liquid Crystallinity Combining Colloidal and Molecular Order in Zwitterionic Molecules. ACS Omega 2023, 8 (42), 39345–39353. 10.1021/acsomega.3c04914. 37901556 PMC10601052

[ref40] Flory P. J.; Vrij A. Melting Points of Linear-Chain Homologs. The Normal Paraffin Hydrocarbons. J. Am. Chem. Soc. 1963, 85 (22), 3548–3553. 10.1021/ja00905a004.

[ref41] Jordan E. F.; Feldeisen D. W.; Wrigley A. N. Side-chain crystallinity. I. Heats of fusion and melting transitions on selected homopolymers having long side chains. J. Polym. Sci. A-1 Polym. Chem. 1971, 9 (7), 1835–1851. 10.1002/pol.1971.150090705.

[ref42] Lin J. C. Y.; Huang C.-J.; Lee Y.-T.; Lee K.-M.; Lin I. J. B. Carboxylic acid functionalized imidazolium salts: sequential formation of ionic, zwitterionic, acid-zwitterionic and lithium salt-zwitterionic liquid crystals. J. Mater. Chem. 2011, 21 (22), 8110 10.1039/c1jm10580a.

[ref43] Rondla R.; Lin J. C. Y.; Yang C. T.; Lin I. J. B. Strong tendency of homeotropic alignment and anisotropic lithium ion conductivity of sulfonate functionalized zwitterionic imidazolium ionic liquid crystals. Langmuir 2013, 29 (37), 11779–11785. 10.1021/la402336n. 24010889

[ref44] James J. D.; Spittle J. A.; Brown S. G. R.; Evans R. W. A review of measurement techniques for the thermal expansion coefficient of metals and alloys at elevated temperatures. Meas. Sci. Technol. 2001, 12 (3), R1–R15. 10.1088/0957-0233/12/3/201.

[ref45] van der Lee A.; Dumitrescu D. G. Thermal expansion properties of organic crystals: a CSD study. Chem. Sci. 2021, 12 (24), 8537–8547. 10.1039/D1SC01076J. 34221335 PMC8221191

[ref46] Watanabe R.; Iyoda T.; Yamada T.; Yoshida H. Thermal expansion of liquid crystalline amphiphilic di-block copolymer observed by simultaneous DSC-XRD. J. Therm. Anal. Calorim 2006, 85 (3), 713–717. 10.1007/s10973-005-7632-5.

[ref47] Hsieh Y.-L.; Hu X.-P. Structural transformation of ultra-high modulus and molecular weight polyethylene fibers by high-temperature wide-angle X-ray diffraction. J. Polym. Sci. B Polym. Phys. 1997, 35 (4), 623–630. 10.1002/(sici)1099-0488(199703)35:4<623:Aid-polb10>3.0.Co;2-i.

[ref48] Cholakova D.; Denkov N. Polymorphic phase transitions in triglycerides and their mixtures studied by SAXS/WAXS techniques: In bulk and in emulsions. Adv. Colloid Interface Sci. 2024, 323, 103071 10.1016/j.cis.2023.103071. 38157769

