# Supplementary material for: Complex Self-Organization in n-Alkylammonium Sulfobetaine Zwitterions with High Thermal Stabilities and High Expansion Coefficients
Source: Langmuir. 2025 Feb 11;41(7):4422–34. doi: 10.1021/acs.langmuir.4c02892 (PMC11875396; doi:10.1021/acs.langmuir.4c02892)
Supplement: Supplementary file 1 — la4c02892_si_001.pdf [file la4c02892_si_001.pdf]

Supplementary Information

Complex Self-Organization in n-Alkylammonium Sulfobetaine Zwitterions with High Thermal Stabilities and High Expansion Coefficients

Alyna Lange[1], Lea Holtzheimer[1], Coby Clarke[2], Andreas F. Thünemann[3], Andreas Taubert[1]

[1] Institute of Chemistry, University of Potsdam, Karl-Liebknecht-Straße 24-25, D-14476 Potsdam-Golm, Germany; alylange@uni-potsdam.de, andreas.taubert@uni-potsdam.de
[2] GSK Carbon Neutral Laboratory, Jubilee Campus, The University of Nottingham, Nottingham, NG7 2GA, UK
[3] Bundesanstalt für Materialforschung und –prüfung (BAM), Unter den Eichen 87, D-12205 Berlin, Germany

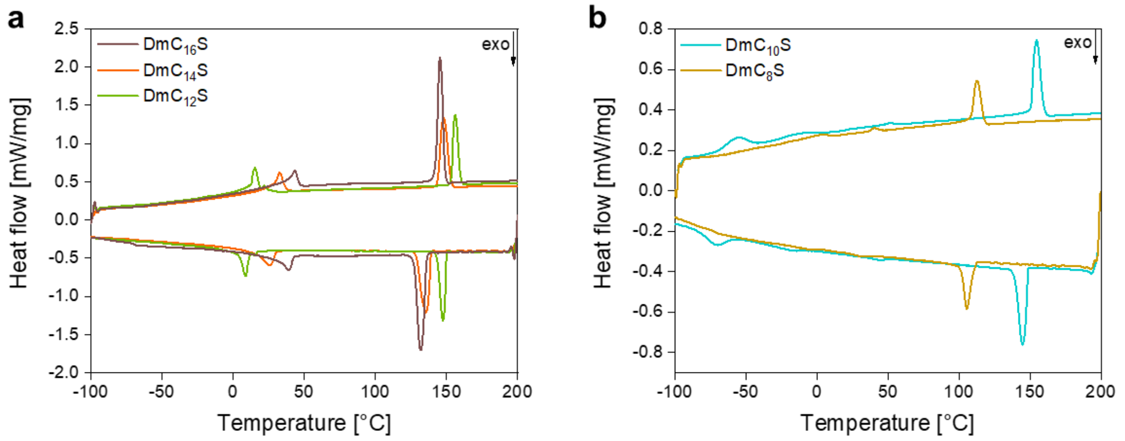

Figure S1: DSC data of 2nd heating and cooling runs: **a)** longer chained ZIs, **b)** shorter chained ZIs.

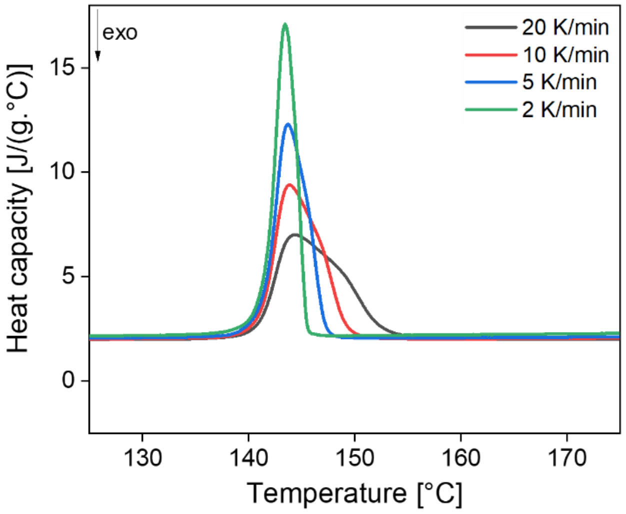

Figure S2: DSC data of heating runs for $DmC_{16}S$ at different heating rates.

Table S1: Water content ZIs according to elemental analysis

	ratio ZI molecule : water molecule
**$DmC_{16}S$**	6 : 1
**$DmC_{14}S$**	2 : 1
**$DmC_{12}S$**	3 : 1
**$DmC_{10}S$**	6 : 1
**DmC_8S**	5 : 1

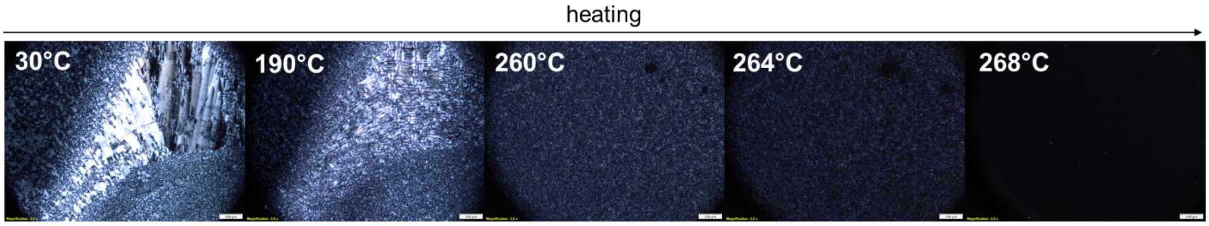

Figure S3: POM images of $DmC_{10}S$.

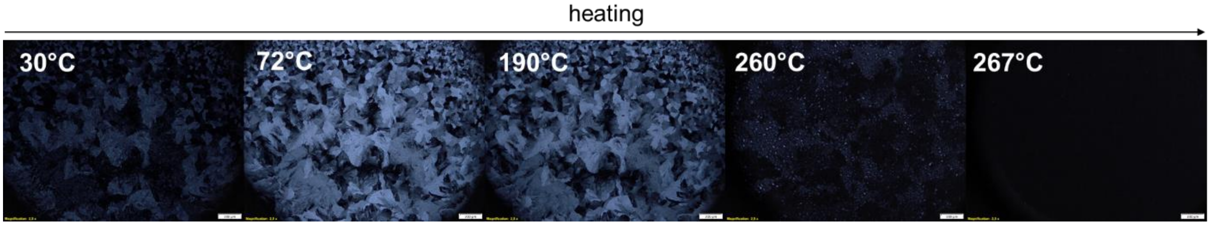

Figure S4: POM images of $DmC_{12}S$.

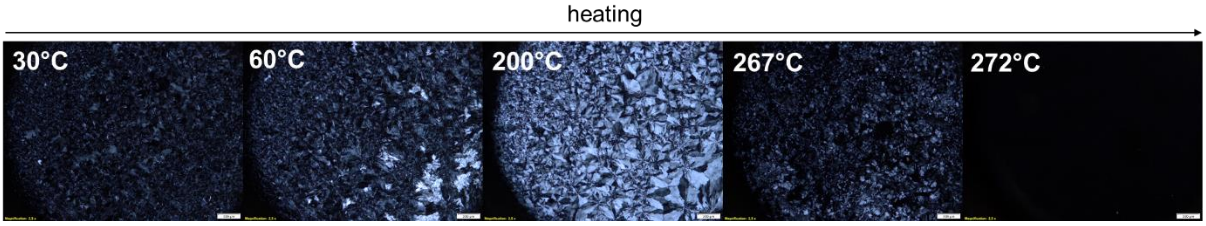

Figure S5: POM images of $DmC_{14}S$.

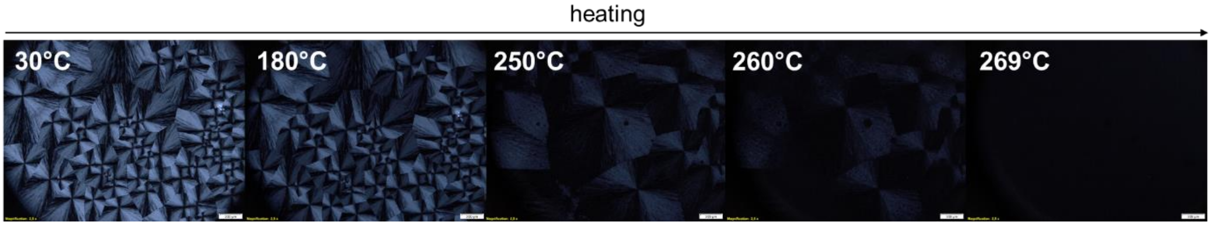

Figure S6: POM images of $DmC_{16}S$.

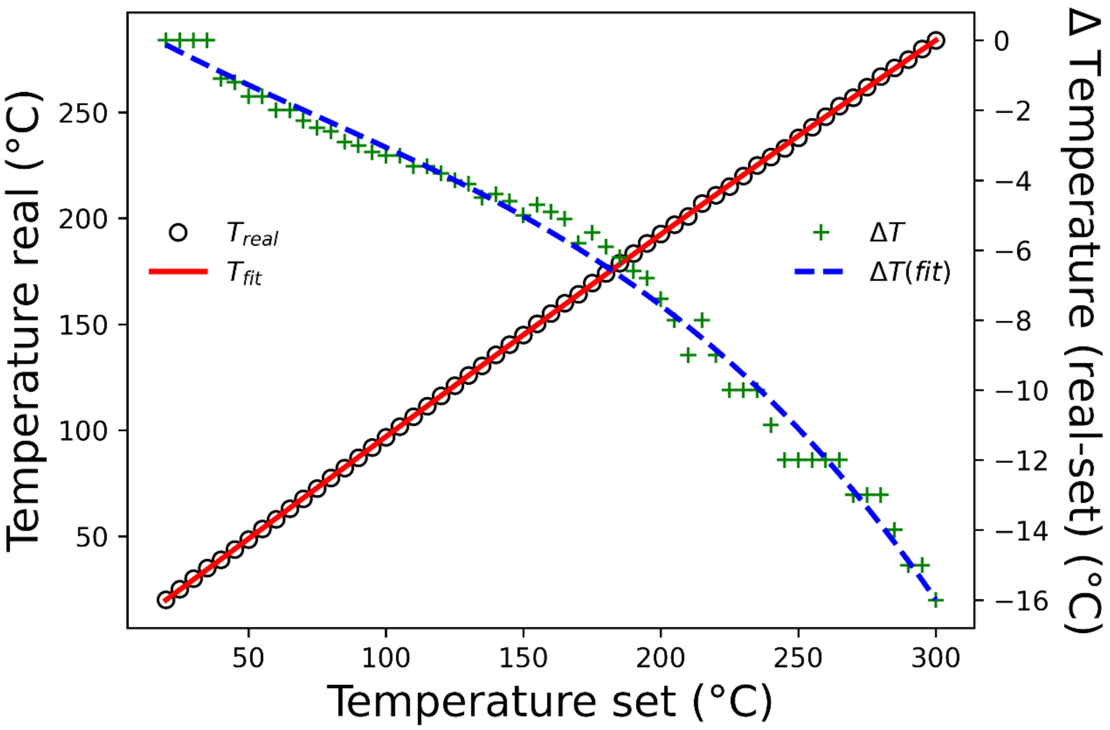

Figure S7: Real (measured) temperature at sample position as a function of the set temperature of the TCS 300 Temperature Control Unit (black circles) and a polynomial curve fit employing $a + b\,x + c\,x^2 + d\,x^3$, with $a = 0.72$, $b = 0.95$, $c = 1.21$ and $d = -5.27$ (red solid line). Right y-axis: The difference between real temperature and set temperature, and the difference resultant from the curve fit (green crosses and blue dashed line, respectively). The maximum difference between real temperature and set temperature is -16°C, i.e., the maximum set temperature of 300°C corresponds to a real temperature of 284°C.

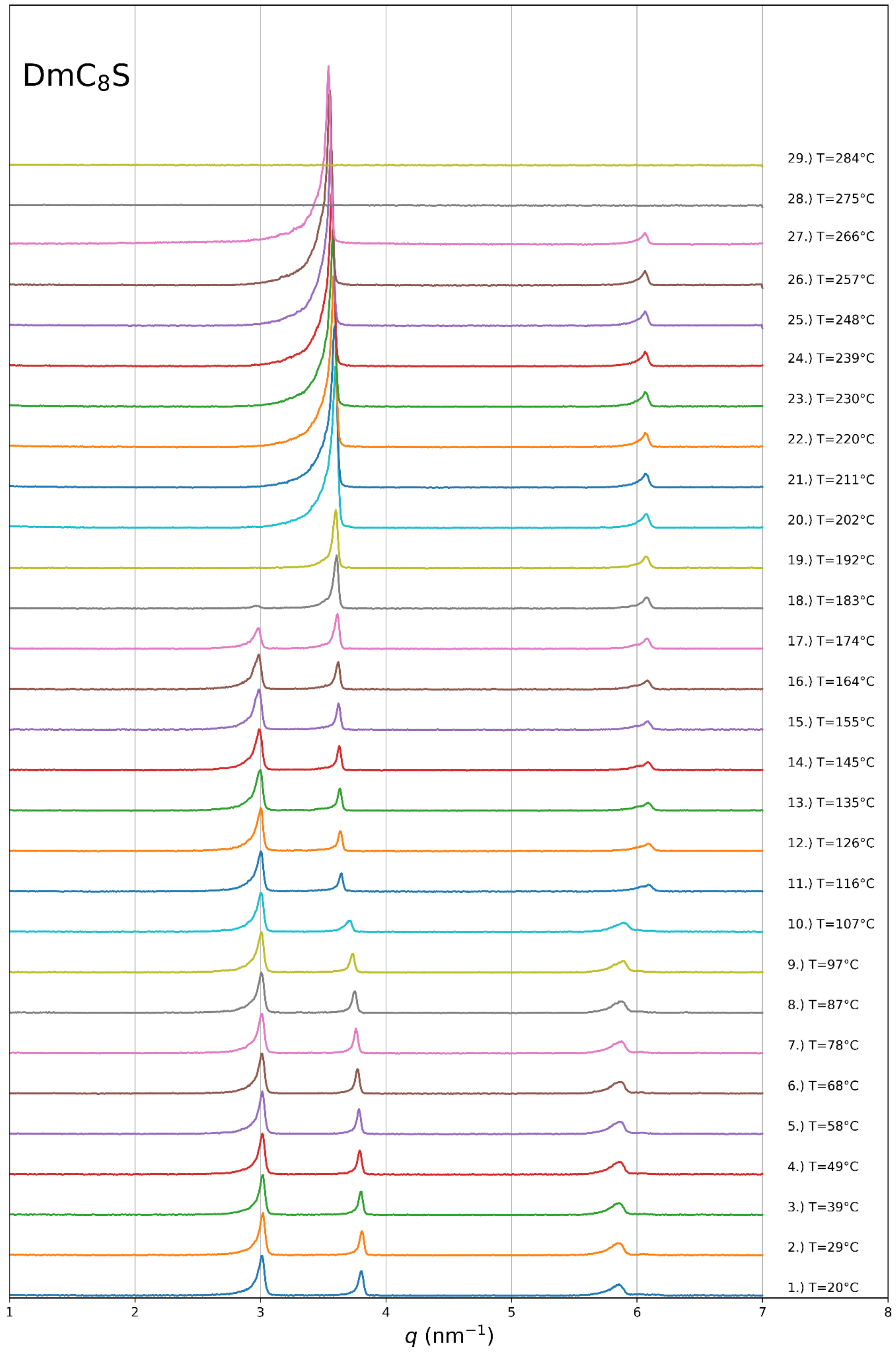

Figure S8: Temperature-dependent SAXS data profiles of DmC_8S.

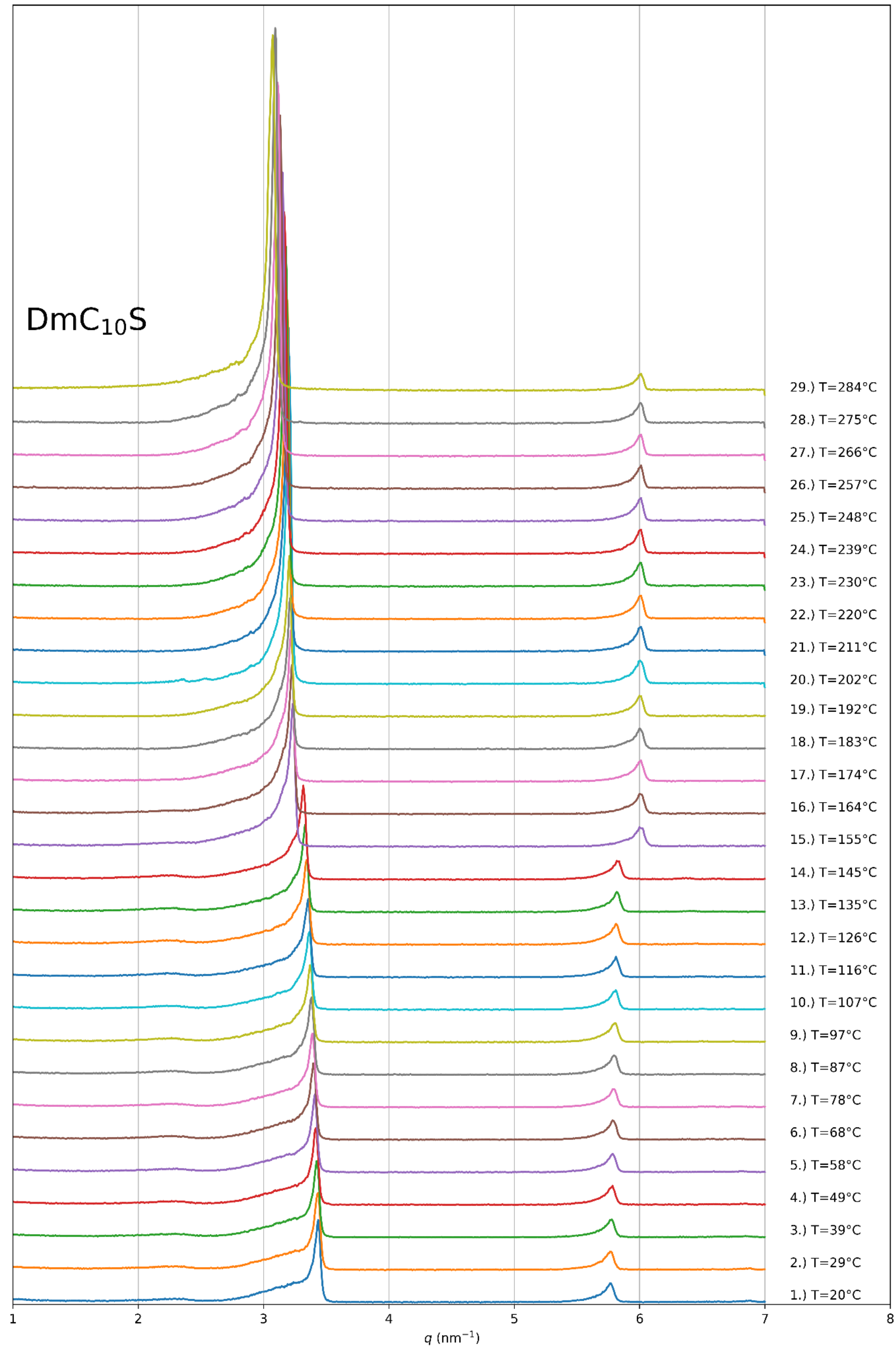

Figure S9: Temperature-dependent SAXS data profiles of $DmC_{10}S$.

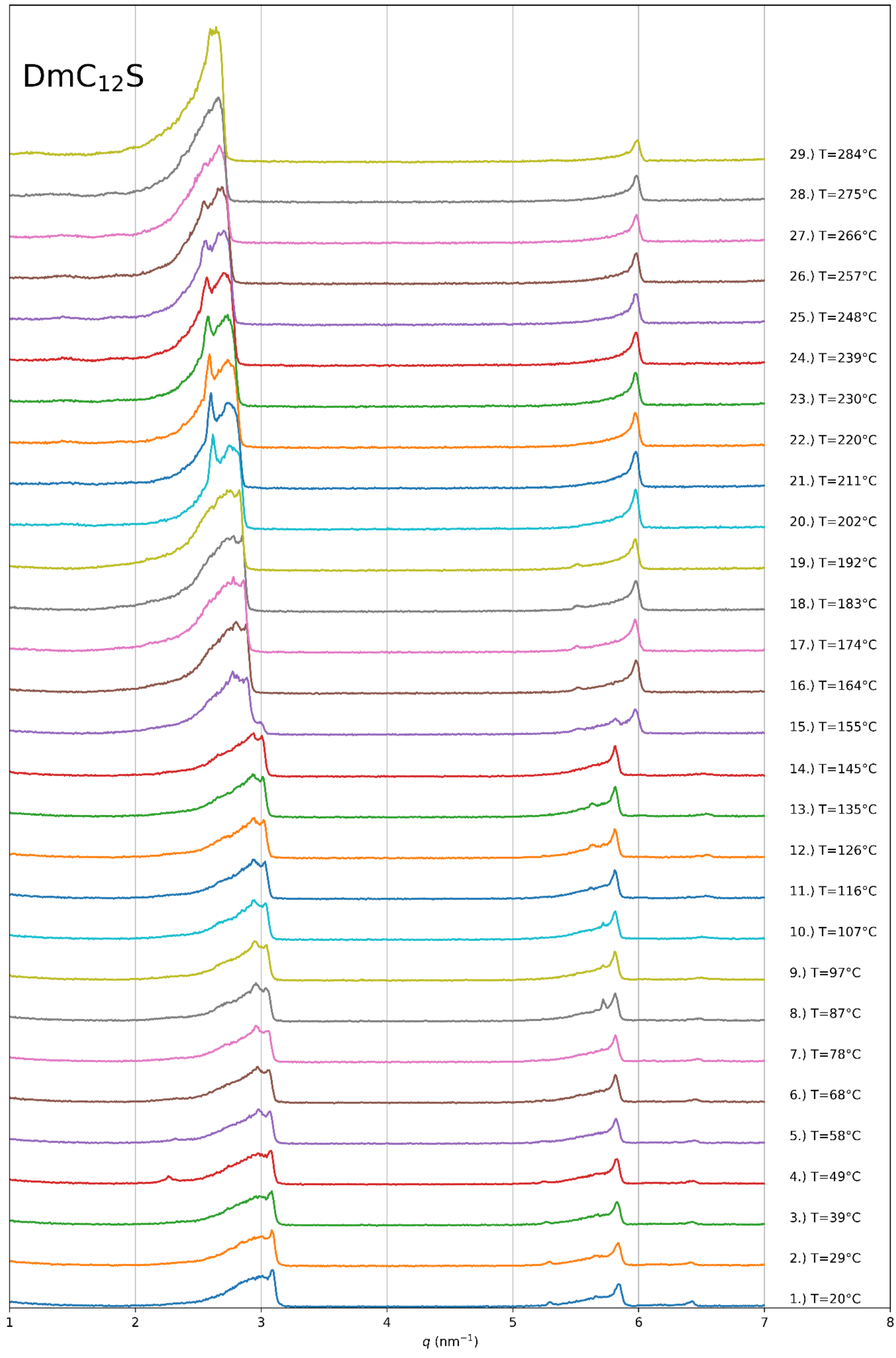

Figure S10: Temperature-dependent SAXS data profiles of $DmC_{12}S$.

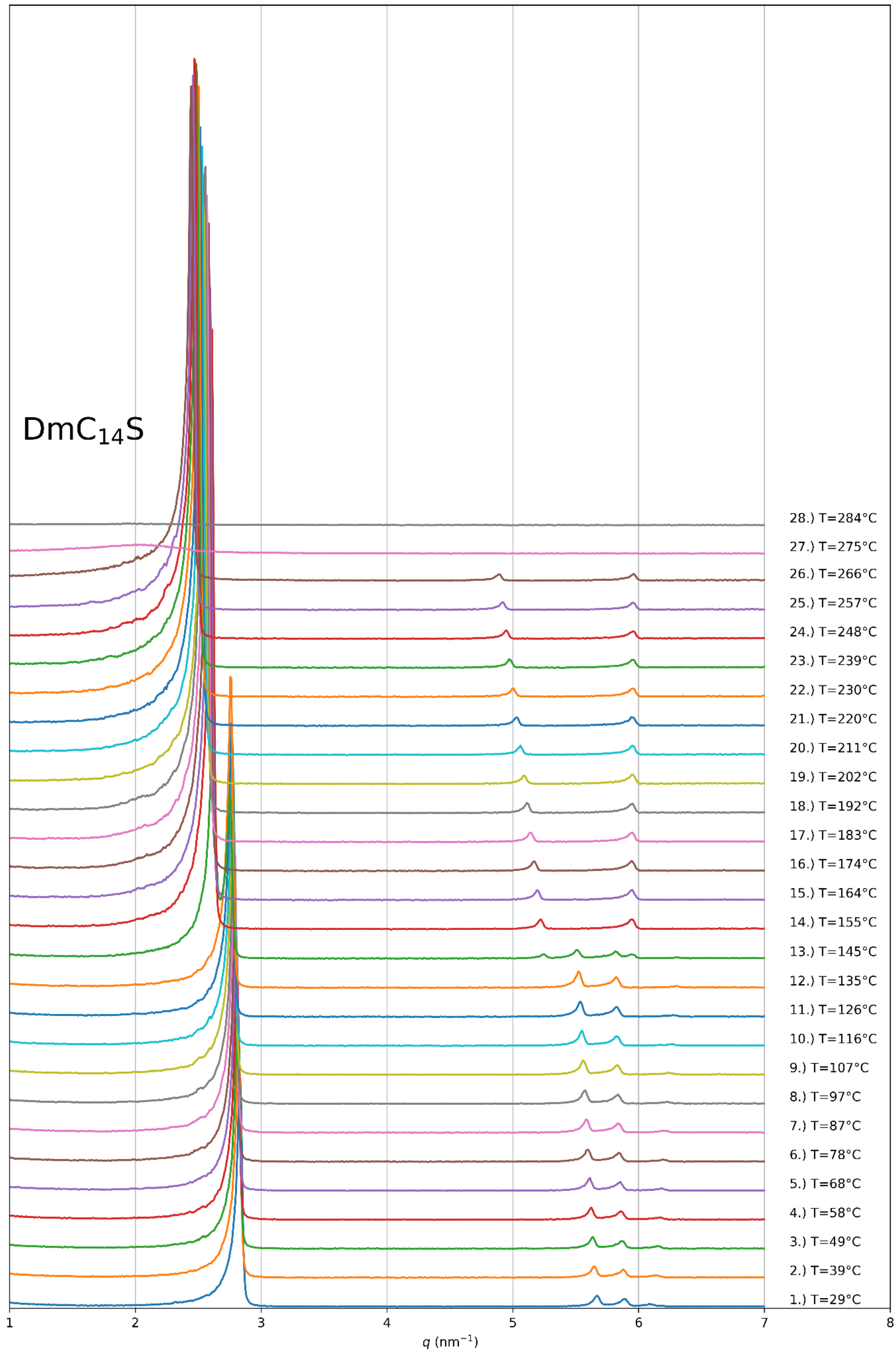

Figure S11: Temperature-dependent SAXS data profiles of $DmC_{14}S$.

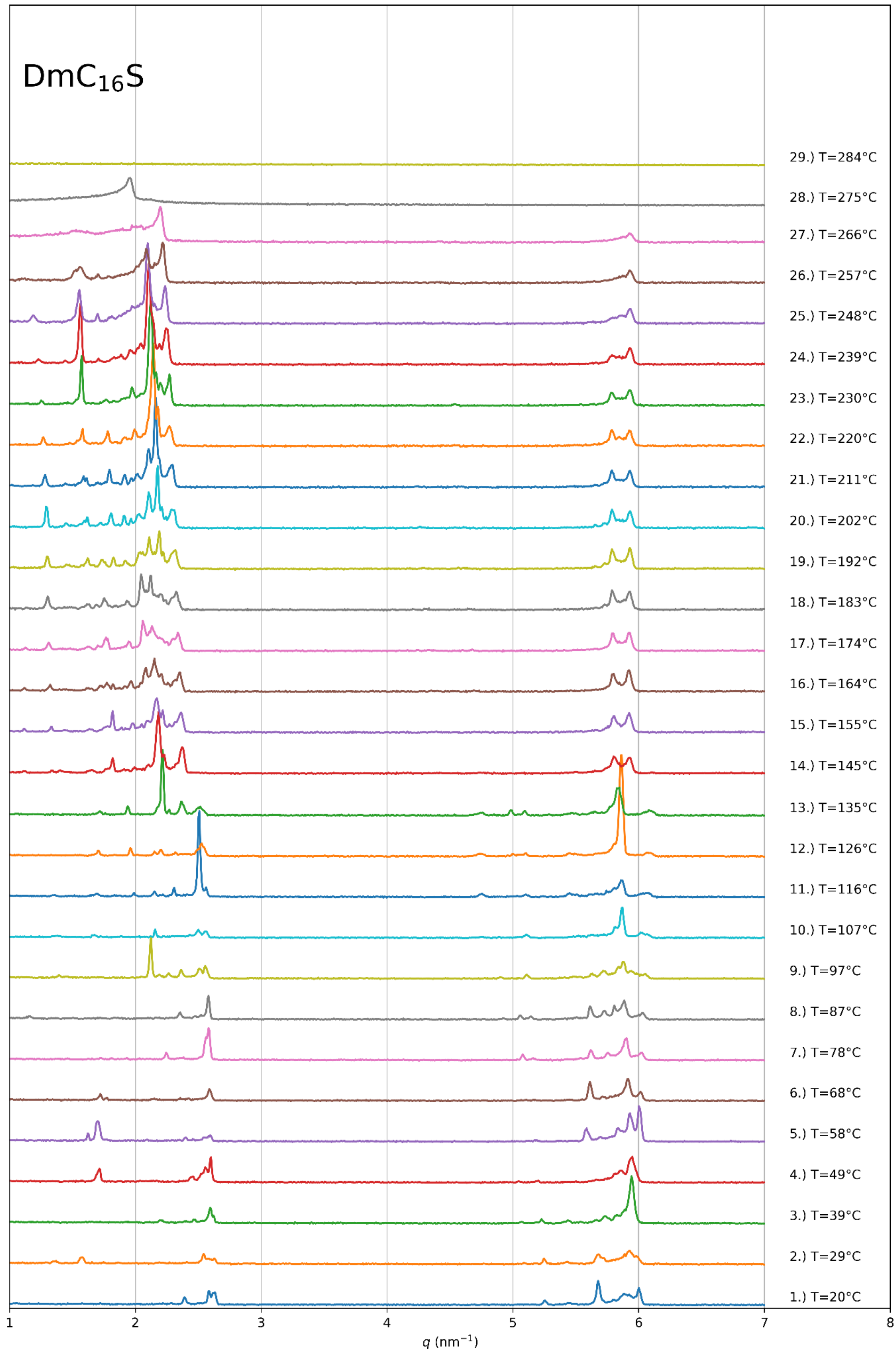

Figure S12: Temperature-dependent SAXS data profiles of $DmC_{16}S$.

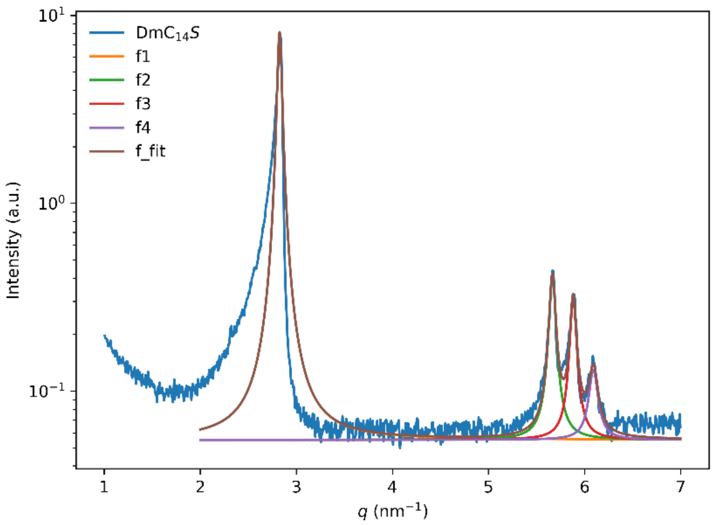

Figure S13: SAXS data of the $DmC_{14}S$ and approximations of the peak utilizing four Lorentzian peak profiles. The temperature was 20°C.

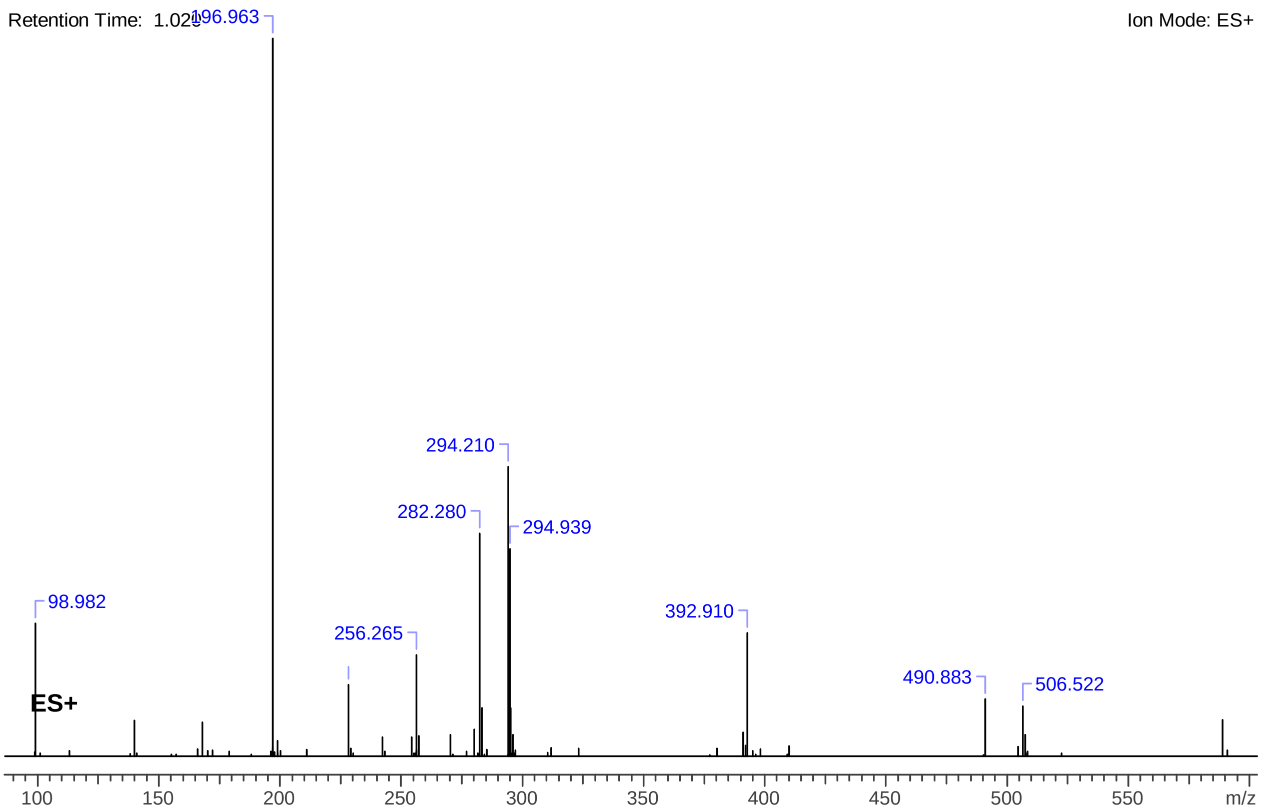

Figure S14: ESI spectrum for $DmC_{10}S$.

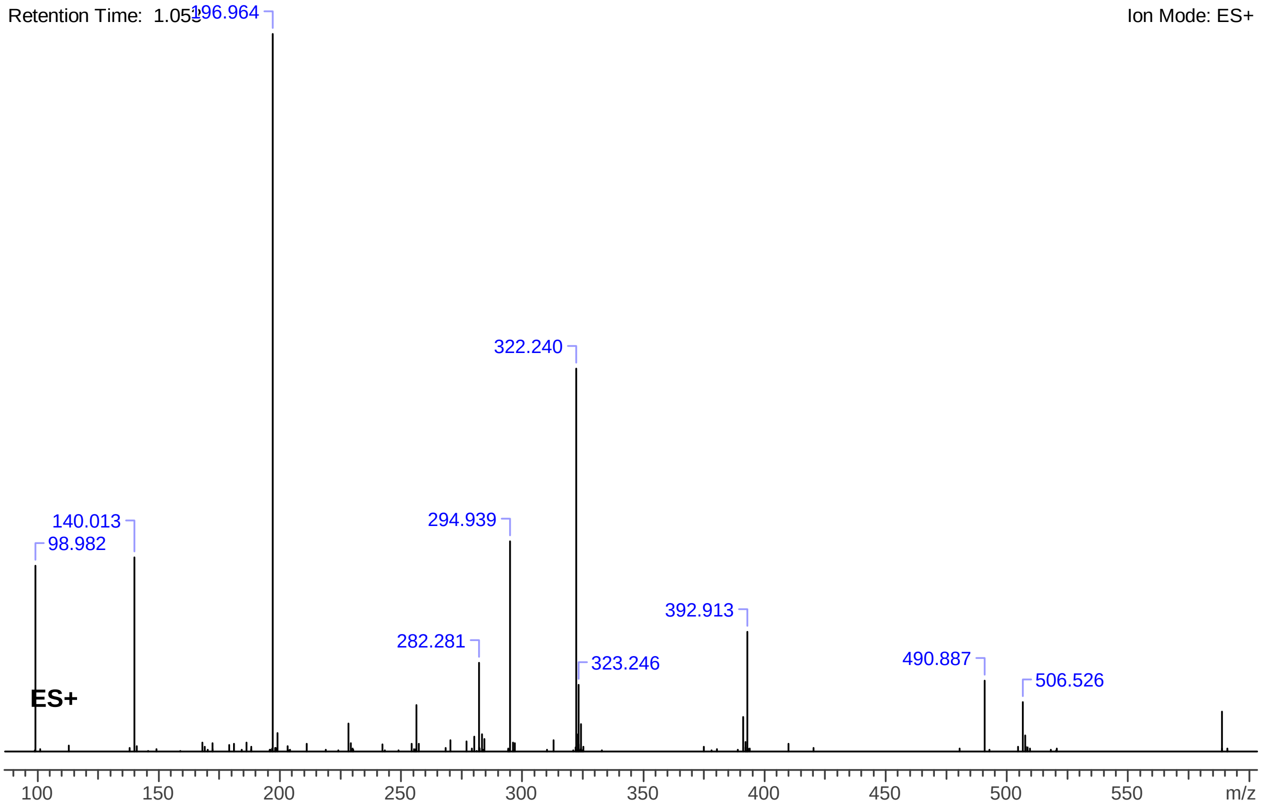

Figure S15: ESI spectrum for $DmC_{10}S$.

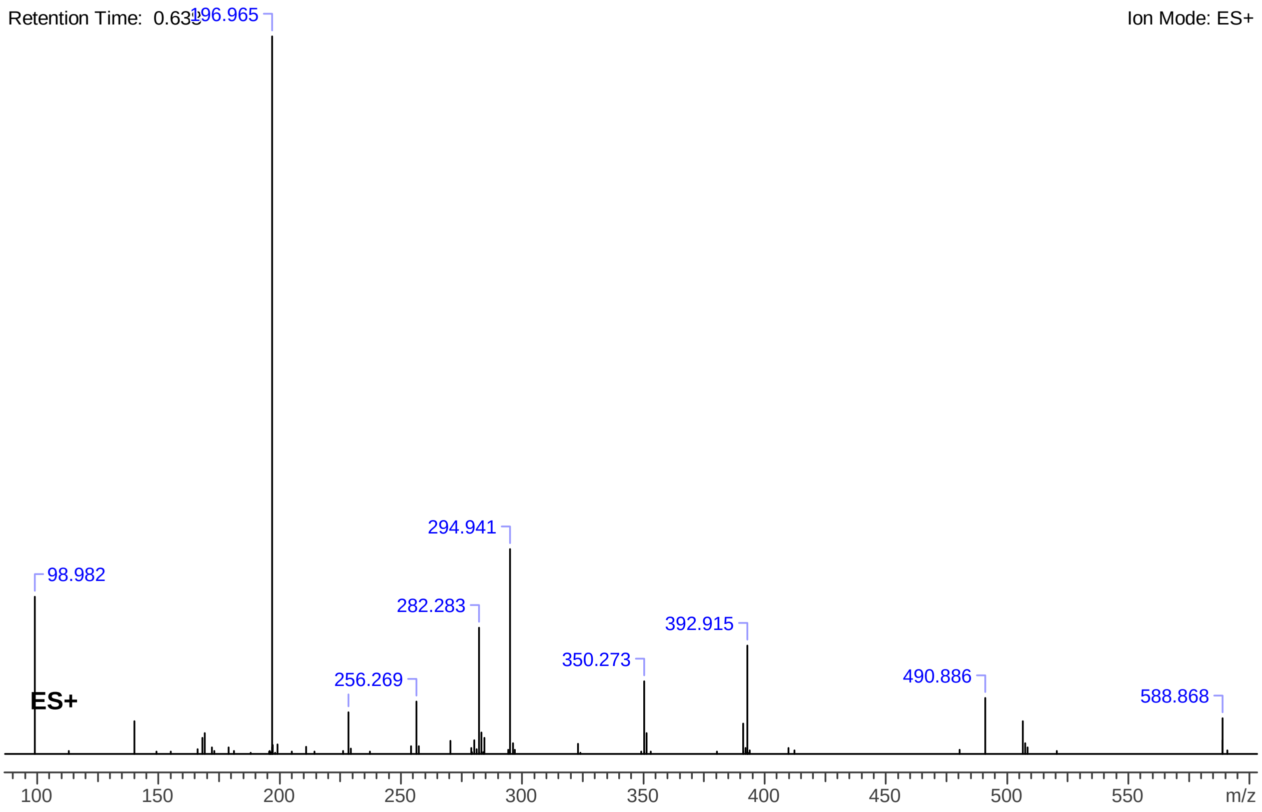

Figure S16: ESI spectrum for $DmC_{12}S$.

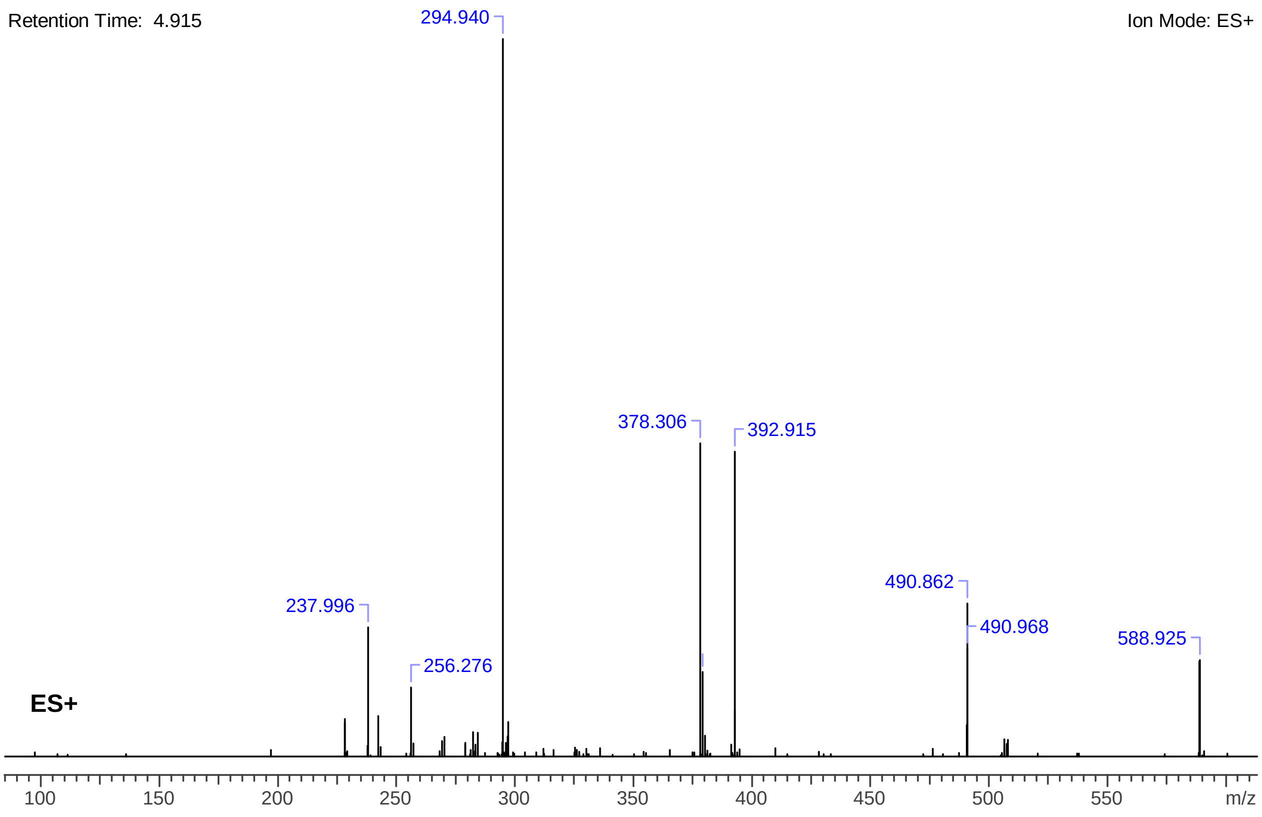

Figure S17: ESI spectrum for $DmC_{14}S$.

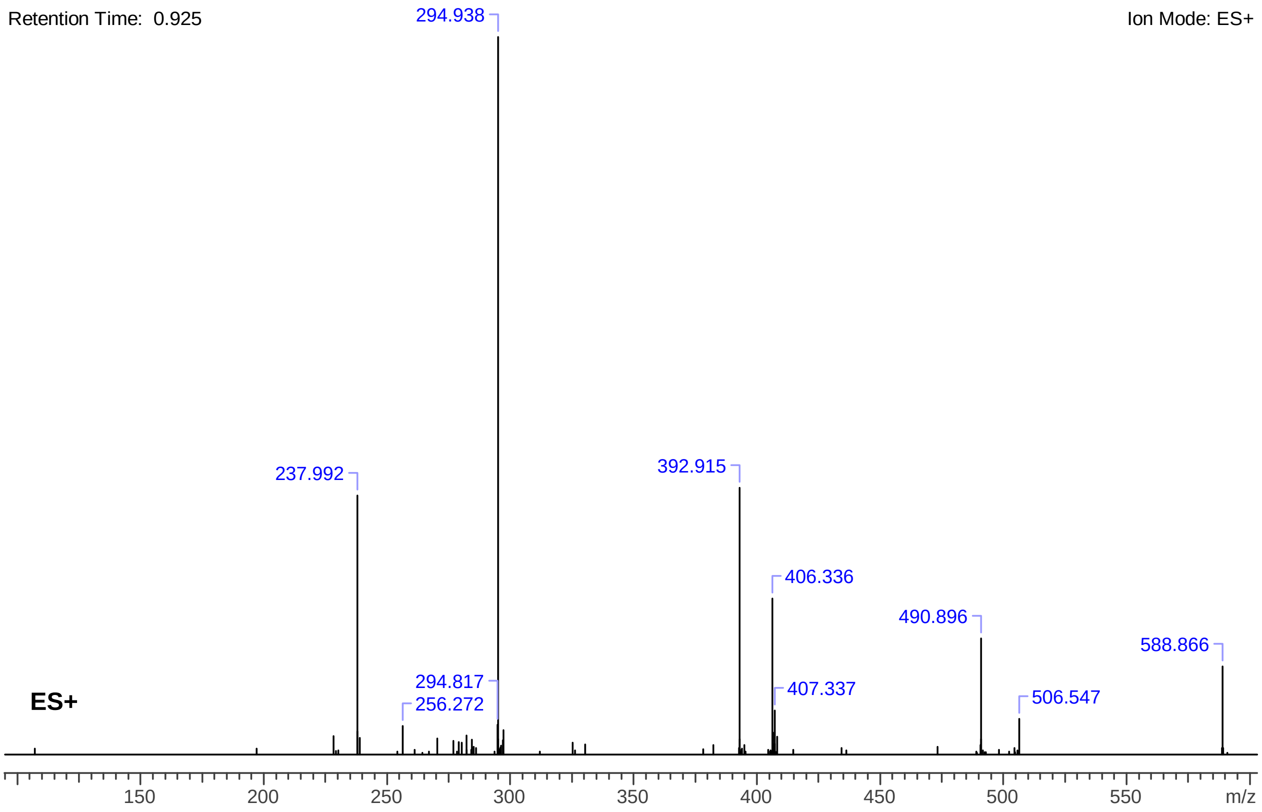

Figure S18: ESI spectrum for $DmC_{16}S$.

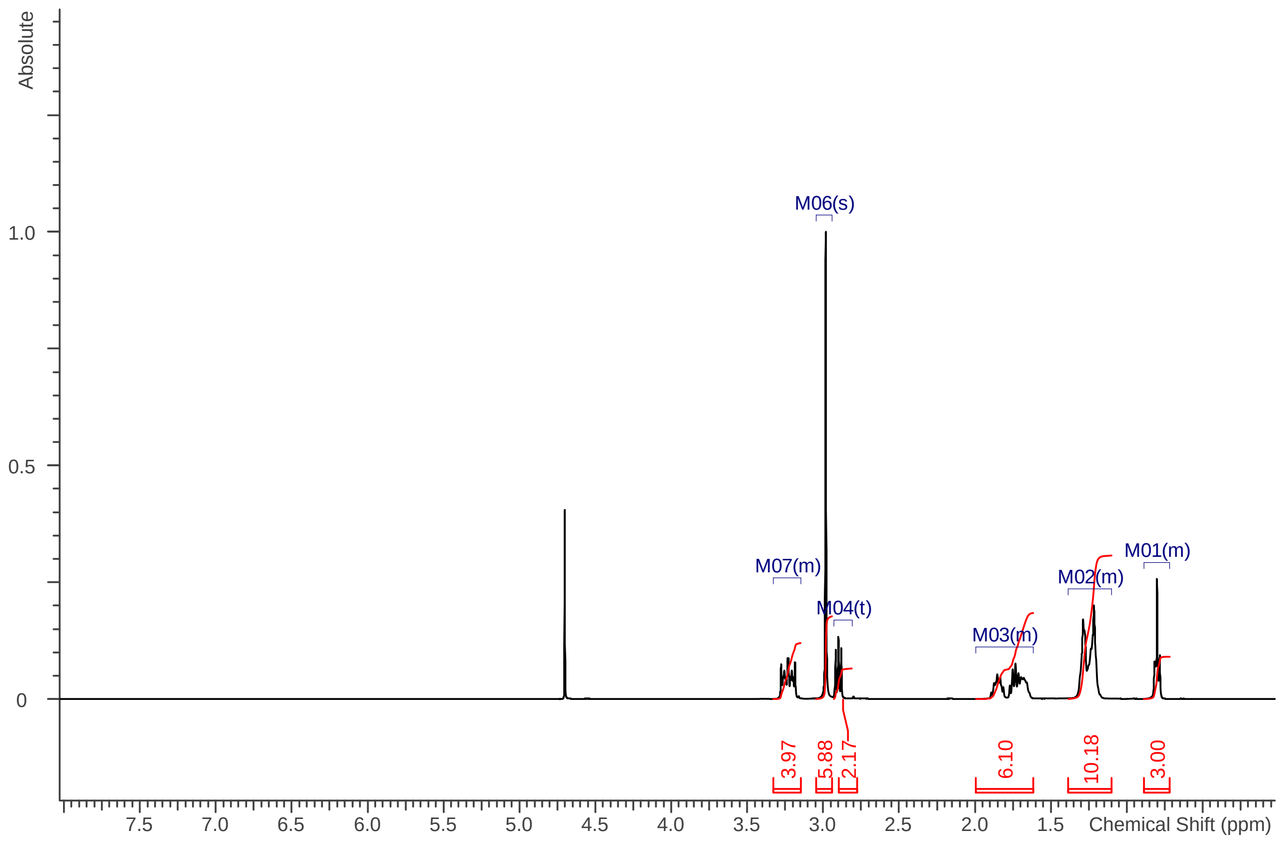

Figure S19: ^{1}H-NMR spectrum for DmC_8S.

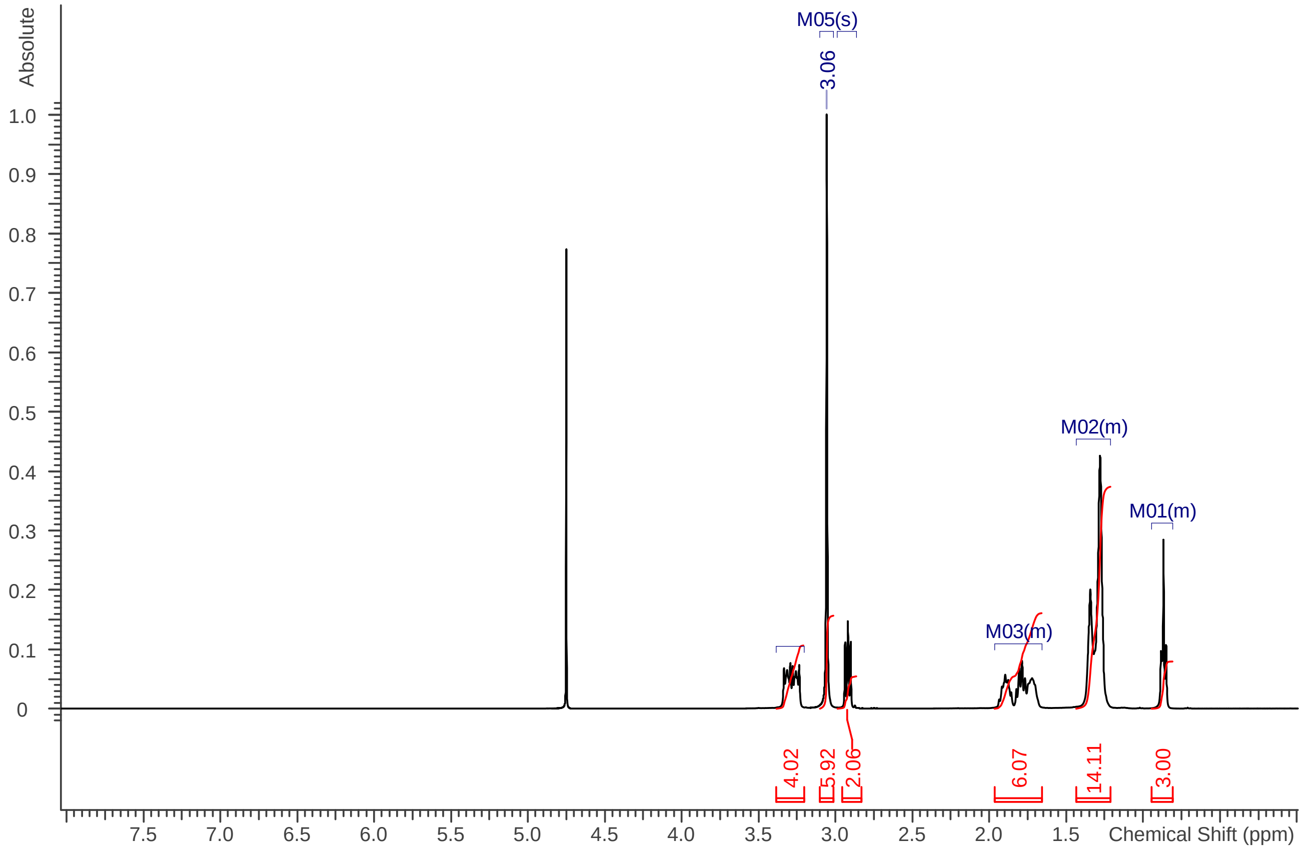

Figure S20: ^{1}H-NMR spectrum for $DmC_{10}S$.

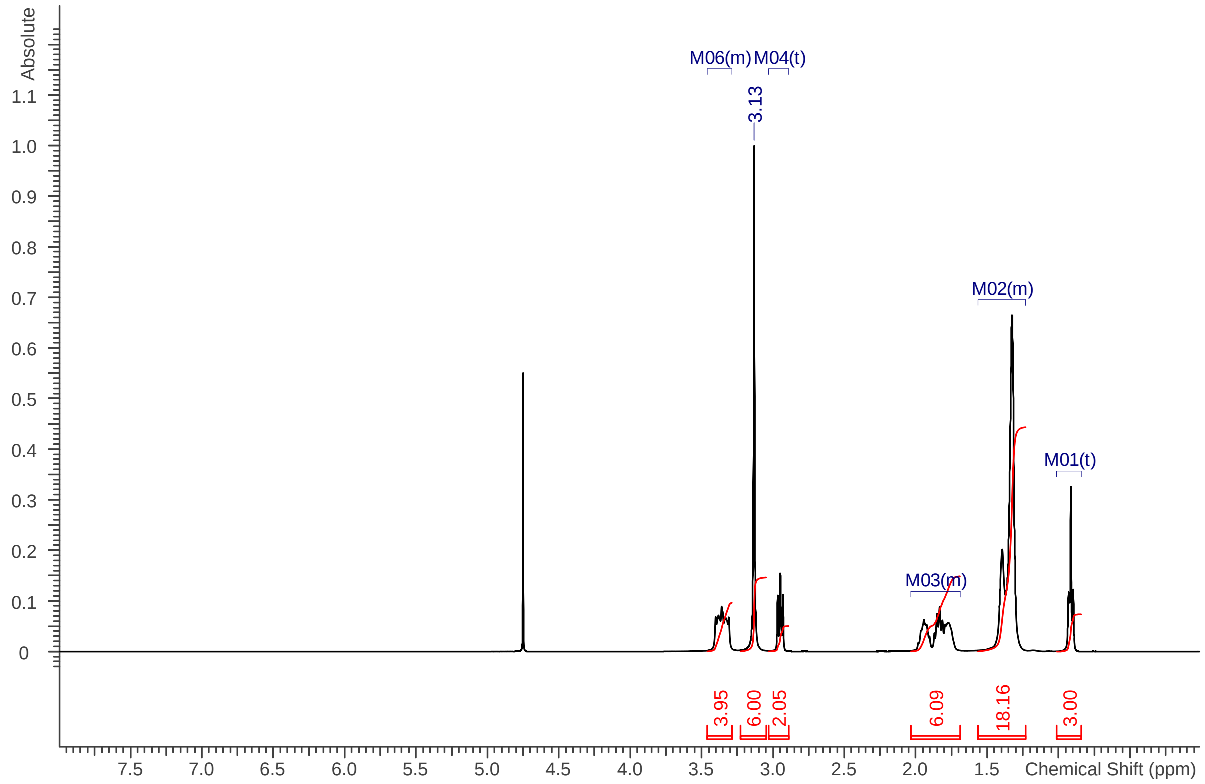

Figure S21: ^{1}H-NMR spectrum for $DmC_{12}S$.

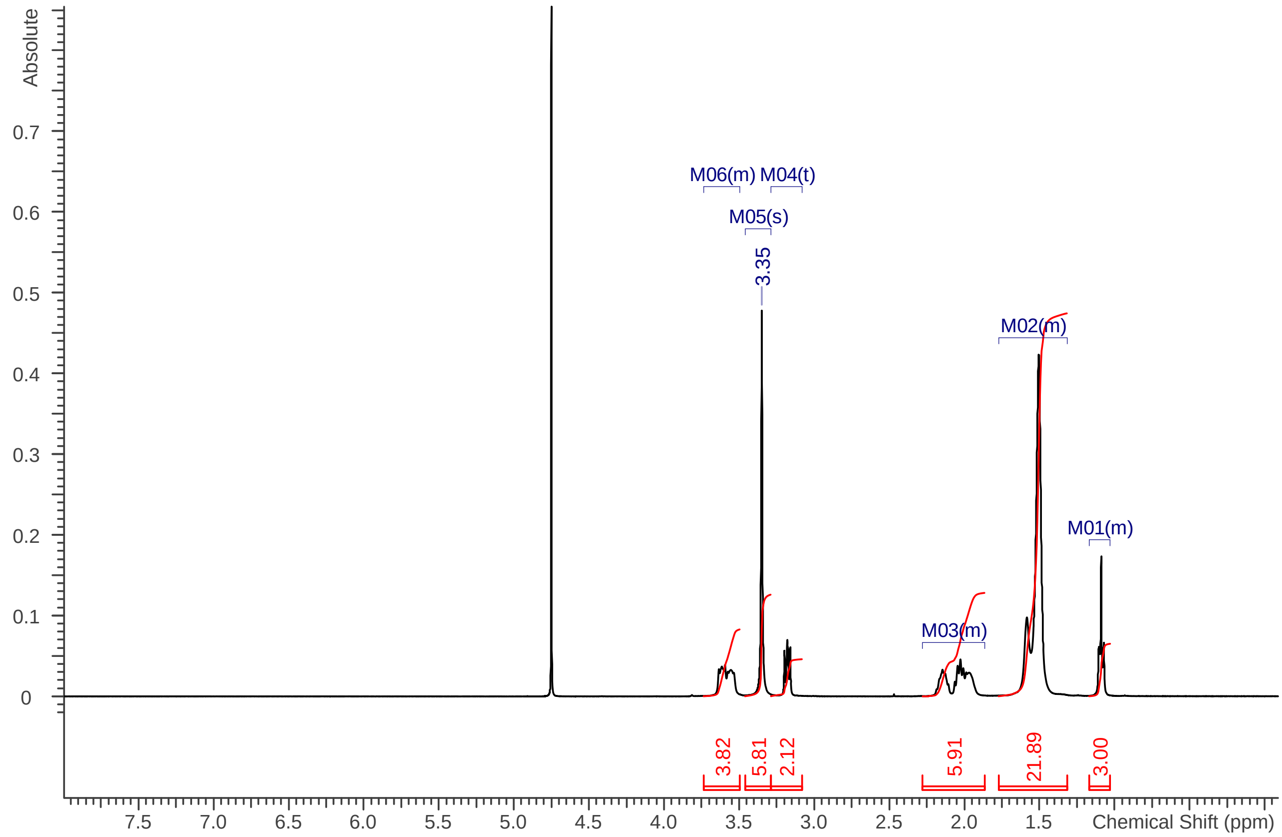

Figure S22: ^{1}H-NMR spectrum for $DmC_{14}S$.

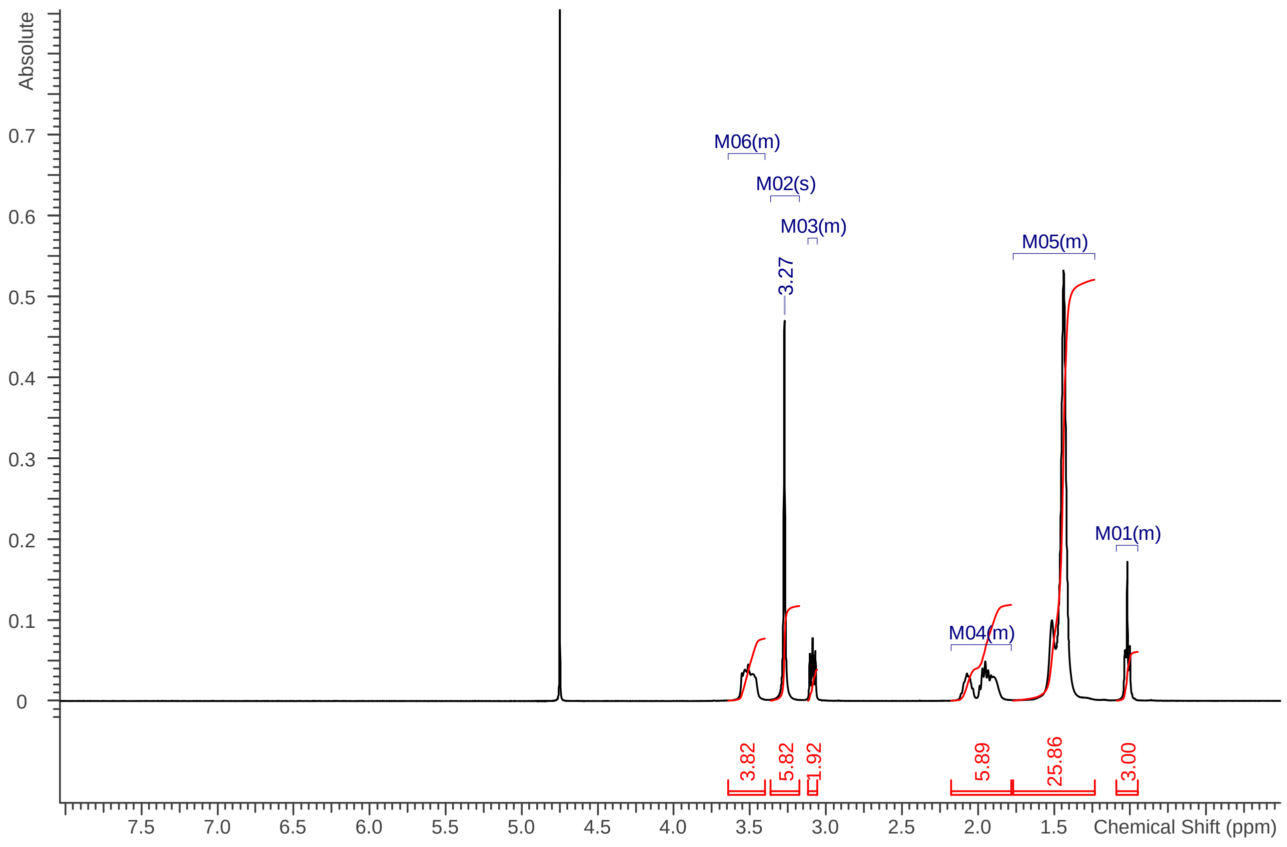

Figure S23: ^{1}H-NMR spectrum for $DmC_{16}S$.
